# Disruption of Z-Disc Function Promotes Mechanical Dysfunction in Human Myocardium: Evidence for a Dual Myofilament Modulatory Role by Alpha-Actinin 2

**DOI:** 10.3390/ijms241914572

**Published:** 2023-09-26

**Authors:** Michelle Rodriguez Garcia, Jeffrey Schmeckpeper, Maicon Landim-Vieira, Isabella Leite Coscarella, Xuan Fang, Weikang Ma, Payton A. Spran, Shengyao Yuan, Lin Qi, Aida Rahimi Kahmini, M. Benjamin Shoemaker, James B. Atkinson, Peter M. Kekenes-Huskey, Thomas C. Irving, Prescott Bryant Chase, Björn C. Knollmann, Jose Renato Pinto

**Affiliations:** 1 Biomedical Sciences, Florida State University, Tallahassee, FL 32306, USA; 2 Department of Medicine, Division of Cardiovascular Medicine, Vanderbilt University Medical Center, Nashville, TN 37232, USA; 3 Department of Cell & Molecular Physiology, Loyola University, Chicago, IL 60660, USA; 4 BioCAT, Department of Biological Sciences, Illinois Institute of Technology, Chicago, IL 60616, USA; 5 Department of Biological Science, Florida State University, Tallahassee, FL 32306, USA; 6 Department of Nutrition and Integrative Physiology, Florida State University, Tallahassee, FL 32306, USA; aida.rahimikahmini@med.fsu.edu; 7 Department of Pathology, Vanderbilt University Medical Center, Nashville, TN 37232, USA

**Keywords:** *ACTN2*, α-actinin 2, Z-disc, cardiomyopathy, muscle mechanics

## Abstract

The *ACTN2* gene encodes α-actinin 2, located in the Z-disc of the sarcomeres in striated muscle. In this study, we sought to investigate the effects of an *ACTN2* missense variant of unknown significance (p.A868T) on cardiac muscle structure and function. Left ventricular free wall samples were obtained at the time of cardiac transplantation from a heart failure patient with the *ACTN2* A868T heterozygous variant. This variant is in the EF 3–4 domain known to interact with titin and α-actinin. At the ultrastructural level, *ACTN2* A868T cardiac samples presented small structural changes in cardiomyocytes when compared to healthy donor samples. However, contractile mechanics of permeabilized *ACTN2* A868T variant cardiac tissue displayed higher myofilament Ca^2+^ sensitivity of isometric force, reduced sinusoidal stiffness, and faster rates of tension redevelopment at all Ca^2+^ levels. Small-angle X-ray diffraction indicated increased separation between thick and thin filaments, possibly contributing to changes in muscle kinetics. Molecular dynamics simulations indicated that while the mutation does not significantly impact the structure of α-actinin on its own, it likely alters the conformation associated with titin binding. Our results can be explained by two Z-disc mediated communication pathways: one pathway that involves α-actinin’s interaction with actin, affecting thin filament regulation, and the other pathway that involves α-actinin’s interaction with titin, affecting thick filament activation. This work establishes the role of α-actinin 2 in modulating cross-bridge kinetics and force development in the human myocardium as well as how it can be involved in the development of cardiac disease.

## 1. Introduction

Alpha-actinin (α-actinin) is an F-actin-crosslinking protein and a member of the actin-binding family or spectrin superfamily of proteins that also includes spectrins and dystrophin proteins [1,2]. Humans have four α-actinin genes that encode for multiple protein products through alternative splicing. These protein products are classified as muscle (or calcium insensitive) and non-muscle cytoskeletal (or calcium-sensitive) proteins [1,3,4]. This study focuses on the cardiac muscle isoform of α-actinin, particularly α-actinin 2 encoded by *ACTN2*, which is 95% conserved across species.

In humans, the Z-disc in striated muscles is mainly comprised of α-actinin 2 [1,5,6], whereas α-actinin 3 is only expressed in fast-twitch skeletal muscle fibers [1,7]. Alpha-actinin 2 and 3 form crosslinks with actin in thin filaments, establishing a lattice-like structure that permits longitudinal force transmission from one sarcomere to the next [1,8]. We propose one communication pathway (CP1), where α-actinin 2 in the Z-disc directly interacts with actin to modulate thin filament regulation. In addition to playing a pivotal role in the structure of muscle, α-actinin 2 and 3 have been involved in the assembly of the myofilaments [1,9]. Some studies report that α-actinin 2 might also have a role as a stretch sensor in the sarcomere, implying that this protein could also participate in tension-sensing and signal transduction of the sarcomere [4]. This suggests a second communication pathway (CP2) where α-actinin 2 interacts with titin and modulates the activation state of the thick filaments. Lastly, α-actinin 2 has been involved in myocyte myogenesis, in which depletion of α-actinin 2 can alter the structure of the sarcomere and lead to disease [9].

Structurally, all spectrin superfamily proteins have an N-terminal actin-binding domain (ABD). The ABD is formed by two calponin homology (CH) domains. Four spectrin repeats (SR) form a central rod that permits flexibility and enables antiparallel homodimer formation that is crucial for α-actinin’s role in the Z-disc [10]. The C-terminus contains a calmodulin (CaM)-like domain that is formed by four EF-hands, EF 1–2 and EF 3–4 [1,4,11].

Striated muscle generates active force when stimulated and experiences passive force when relaxed. Active force production is due to the cyclic interactions of actomyosin cross-bridges that form between thin and thick filaments in the presence of elevated cytoplasmic Ca^2+^. Dimers of α-actinin within the Z-discs on opposite sides of the sarcomere crosslink thin filaments and titin [12]. The CaM-like domain of α-actinin interacts with titin by the Z-repeats [13]. The muscle isoforms of α-actinin 2 and 3 bind phosphatidylinositol 4,5-biphosphate (PiP2). Previous studies have shown that α-actinin bound to PiP2 affects the polymerization and depolymerization of actin. Muscular and non-muscular isoforms of α-actinin interact extensively with PiP2, stimulating actin bundling, suggesting that alterations in PiP2 or α-actinin might affect the function of actin filaments [14]. In addition, the binding of PiP2 by α-actinin enables the interaction of the EF 3–4 hands with titin, particularly with Z-repeat 7, thus affecting the regulation of crosslinks [12,15]. α-actinin 2 is crucial for the structural organization and function of striated muscle by playing fundamental roles not just in anchoring myofilament proteins in the sarcomere but also in regulating ion channels and gene expression [16].

Variants of *ACTN2* are associated with the development of restrictive cardiomyopathy (RCM), hypertrophic cardiomyopathy (HCM), and heart failure (HF) [16,17,18]. Multiple lines of evidence have demonstrated that alteration of the structural integrity of sarcomeres and myofibrils leads to pathology [19]. However, the pathogenesis of how α-actinin 2 variants modify sarcomere integrity is still unclear [8]. In this article, we describe mechanical and molecular markers of an *ACTN2* variant located in the EF 3–4 hand of the protein structure and our interpretation of the mechanism by which it might affect the kinetics of muscle contraction. We propose that this *ACTN2* variant in the EF 3–4 hand affects interactions with surrounding sarcomere proteins, including titin. Furthermore, we propose that abnormal interactions between α-actinin 2 and sarcomere proteins may alter the compliance of the Z-disc, leading to dysfunctional actomyosin interactions in the sarcomere and thereby altering cardiac muscle kinetics.

## 2. Results

### 2.1. ACTN2 *A868T* Variant as a Candidate for Cardiac Disease Etiology

**Figure 1:**
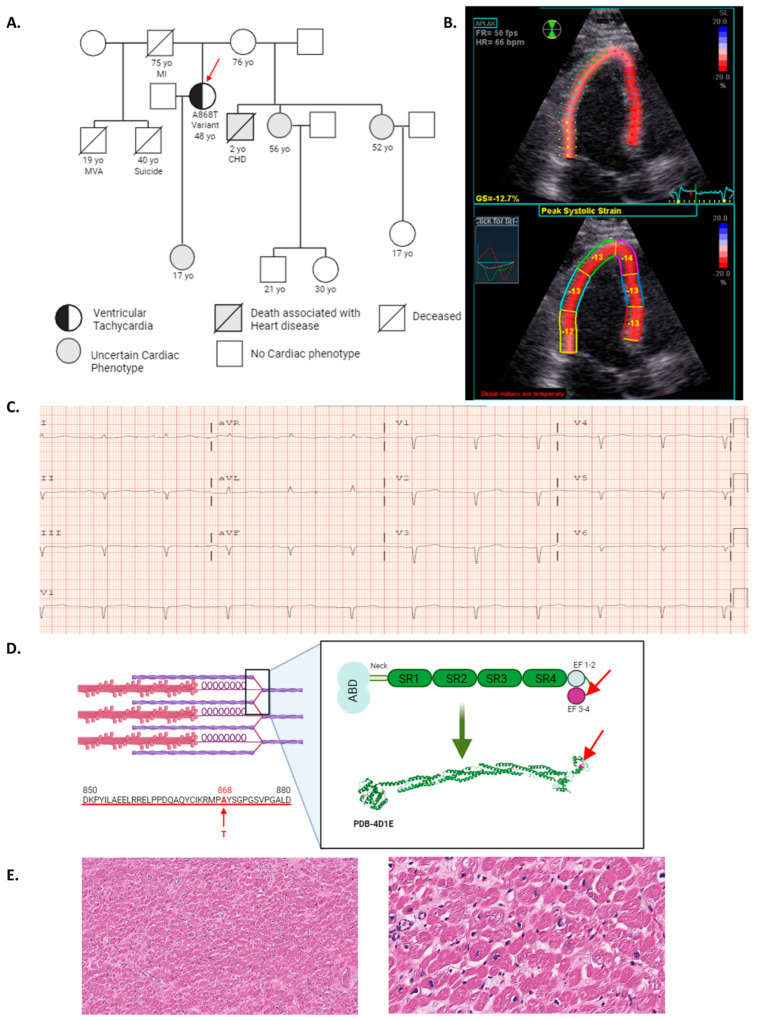
***ACTN2* (α-actinin 2) variant as a strong candidate for diverse cardiac signs and symptoms.** De-identified human free left ventricular wall was obtained from an A868T variant patient after heart transplant. (**A**) Family pedigree of A868T variant patient, red arrow marks proband patient. (MI: myocardial infarction, MVA: motor vehicle accident, CHD: congenital heart disease). (**B**) Representative echocardiographic longitudinal strain of proband patient. (**C**) Presenting electrocardiogram of proband patient. (**D**) Location of the A868T variant in the sarcomere (top left), primary sequence (bottom left), the red arrow shows where the variant is located and the amino acid substitution, schematic (top right), and crystal structure (bottom right) of α-actinin 2. (**E**) Representative H&E stained sections from explanted left ventricle free wall from the proband patient at 10× (left) and 40× (right) magnification.

**Figure A1:**
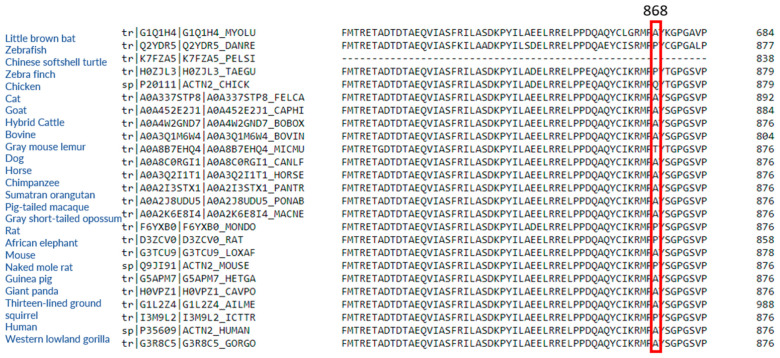
**Sequence of α-actinin 2 EF 3–4 region across 25 different species.** Multi-species sequence alignment of the region where the A868T variant is located. Highlighted by the red box is the amino acid that is mutated in the human patient.

**Figure A2:**
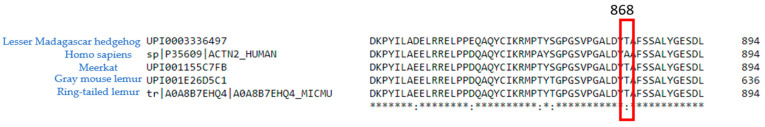
**Sequence of α-actinin 2 EF 3–4 in four small mammals that have threonine (T) at the equivalent residue as to human A868.** Multi-species sequence alignment of the region where the A868T variant is located. Highlighted by the red box is the amino acid that is mutated in the human patient, aligned with four small mammal species that, surprisingly, normally have a threonine (T) residue at that location.

A 48-year-old female patient was evaluated at Vanderbilt University Medical Center with a witnessed out-of-hospital ventricular fibrillation arrest. The patient had no previous cardiac history prior to her arrest. The patient’s family history includes several family members with cardiac symptomology; however, no further genetic studies of the family have been conducted due to inconsistent follow-ups in the patient and family members’ medical histories (pedigree presented in Figure 1A). A transthoracic echocardiogram (Figure 1B) revealed: (1) normal left ventricular ejection fraction estimated to be 50–60% (54–74% is considered normal in the female population [20], (2) stage II diastolic dysfunction, (3) elevated right ventricular systolic pressure calculated as 28 mmHg (normal < 25 mmHg [21], and (4) abnormal global longitudinal strain at −13% (overall population −17.5% [22] consistent with early signs of hypertrophic cardiomyopathy (HCM) and early left ventricular systolic dysfunction. The ECG showed a prolonged PR interval, suggesting an atrioventricular (AV) block type I as well as abnormal R-wave progression (Figure 1C). Her clinical presentation and her cardiopulmonary exercise testing classified her as AHA functional class IV [23,24,25], and the patient subsequently underwent a cardiac transplant. Genetic testing combining cardiac sequencing and deletion/duplication panel reported a heterozygous missense variant of uncertain significance (VUS) in *ACTN2* (p.Ala868Thr (A868T)) (Figure 1D). According to ClinVar, available data regarding this VUS is insufficient to determine the role of this variant in the development of the disease. *ACTN2* encodes for α-actinin 2, which is conserved across species. While the EF3–4 region of α-actinin 2 is highly conserved, the particular amino acid found mutated in the patient (868) is not conserved across species (Figure A1). However, A868T mutates the residue from a hydrophobic to a polar residue, which might be expected to alter protein–protein interactions, resulting in impaired function (Figure A2). In addition to the presence of the *A868T* variant, the genetic test reported two additional heterozygous VUSs. *DSC2* (p.Val739Leu or V739L) and *SCN2B* (p.Gly138Ser or G138S) variants. *DSC2* gene encodes for desmocollin-2, a protein important for desmosome assembly. This variant is associated with arrhythmogenic right ventricular dysplasia (ARVD). Since our patient did not present signs or symptoms of ARVD, this variant was excluded as a possible cause of the patient’s symptoms by her medical team. The *SCN2B* gene encodes for the β 2 subunit of type II voltage-gated sodium channel and is associated with familial atrial fibrillation. The medical team proceeded with a procainamide test, but no abnormality was reported, suggesting that this variant may not contribute to the patient’s symptoms. Histopathological evaluation using hematoxylin and eosin (H&E) staining of sections from the left ventricle free wall demonstrated mild interstitial fibrosis through the myocardium, resembling the appearance and distribution of amyloid infiltration (Figure 1E). Previous studies have described a higher index of myofibrillar disarray associated with *ACTN2* variants [26]. Histological images showed slight myocyte disarray that could be consistent with findings from small-angle X-ray fiber diffraction of muscle, as described below.

### 2.2. Characterization of Sarcomere Alterations in the Patient’s Heart

**Figure 2:**
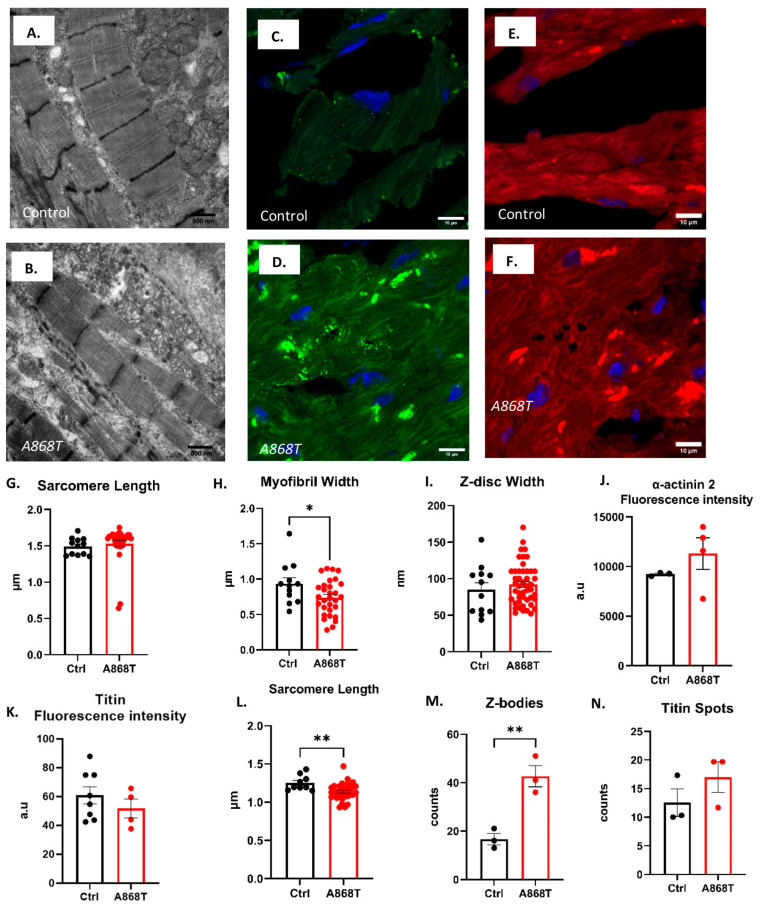
**Ultrastructural analysis of the A868T variant patient heart.** (**A**,**B**) Representative electron microscopy of (**A**) normal and (**B**) A868T variant myocardium. Scale bars are 800 nm. (**C**–**F**) Representative immunofluorescence images with α-actinin 2 (green) and titin (red) antibodies. Scale bars are 10 µm. Additional immunofluorescence images are shown in Supplementary Figures S2 and S3. (**G**) Sarcomere length measured from EM images (5 EM images of the same patient’s heart and 5 EM images of the same donor heart were analyzed). (**H**) Myofibrillar width measured from EM images (5 EM images of the same patient’s heart and 5 EM images of the same donor heart were analyzed). (**I**) Z-disc width measured from EM images (5 EM images of the same patient’s heart and 5 EM images of the same donor heart were analyzed). (**J**,**K**) Antibody fluorescence intensity quantification. (**L**) Sarcomere length measured from immunofluorescence images. (**M**) Z-bodies quantification measured from immunofluorescence images. (**N**) Titin spots measured from immunofluorescence images. Data are shown as mean ± S.E; the data points are technical replicates. * *p* < 0.05. ** *p* < 0.01.

Previous studies have described the general structural changes associated with pathological remodeling. Mechanical stress results in altered signaling pathways leading to abnormal mechano-transduction within the myocytes [27,28,29,30,31]. α-actinin in the Z-disc has close interactions with titin and is associated with sensing sarcomere length (SL) changes [28,30]. In order to quantify sarcomere structural characteristics, we analyzed electron microscopy (EM) images obtained from the control (Figure 2A) and A868T heart (Figure 2B). Our A868T samples had a SL of 1.53 μm, which was not significantly different from the control sample SL of 1.49 μm (Figure 2G). Myofibril width was, however, significantly smaller in our A868T tissue as compared to the control (0.74 μm vs. 0.93 μm, respectively) (Figure 2H) [26,32,33]. Moreover, the Z-disc width was 92 nm in A868T samples, which was not significantly different from our control samples (85 nm) [34] (Figure 2I). These findings suggest that the A868T variant might not affect the structure of α-actinin 2 per se; rather, it impacts its interactions with surrounding sarcomere proteins [35]. Although the overall architecture within the Z-disc appears to be preserved, the A868T variant appears to produce abnormal structural changes elsewhere in the sarcomere that could underlie the clinical findings.

Next, we assessed the structural implications of our A868T variant using immunoassay techniques. Confocal microscopy immunofluorescence images from cryo-sectioned samples of A868T heart tissue and control heart tissue are shown in Figure 2C–F and Figure S1. We observed that α-actinin 2 striations showed higher fluorescence intensity in the A868T variant when compared with the control striations for anti-α-actinin 2 antibodies (Figure 2J), but this increase was not significant [36,37]. We observed that our A868T samples presented a slight but not significant reduction in fluorescence intensity from anti-titin antibodies (Figure 2K) relative to the control (Figure S2). We also evaluated the SL from the immunofluorescence images obtained with confocal microscopy. Sarcomere length was significantly smaller in A868T samples than in control samples, being 1.11 µm in the A868T variant and 1.26 µm in the control (Figure 2L). We speculate that the changes in SL observed between EM and immunofluorescence images are due to specimen preparations. Taken together, our immunoassay results support the notion that the A868T variant in α-actinin 2 alters the structure of the sarcomere and, to some extent, adjusts protein expression as a compensatory mechanism to maintain functional sarcomere organization [37]. It appears, however, that these compensatory mechanisms might fail over time and lead to pathology, as seen in our patients. Z-bodies were abundant in images from our A868T samples [38,39] as well as in images from control samples (Figure 2C,D), with significantly more Z-bodies in our A868T samples (mean of 42 units) when compared with the control (mean of 16 units, Figure 2M) [40]. We propose that the observed higher number of Z-bodies represents a compensatory mechanism to counteract the altered sarcomere structure in the myofibrillogenesis stage induced by the presence of the variant. Previous studies have reported a correlation between the process of cardiac myofibrillogenesis of Z-bodies and the presence of titin aggregates or spots [41,42]. Our group observed that there were no significant changes regarding titin spot production and distribution in our A868T samples (Figure 2N). These results suggest that even though the sarcomere structure is affected to some degree, maturation of the sarcomere might be affected by the A868T variant and that compensatory mechanisms may act to maintain the functionality of the cardiac muscle.

### 2.3. Abnormal Mechanics of Contraction in Cardiac Muscle Preparations Obtained from the Patient’s Heart

**Figure 3:**
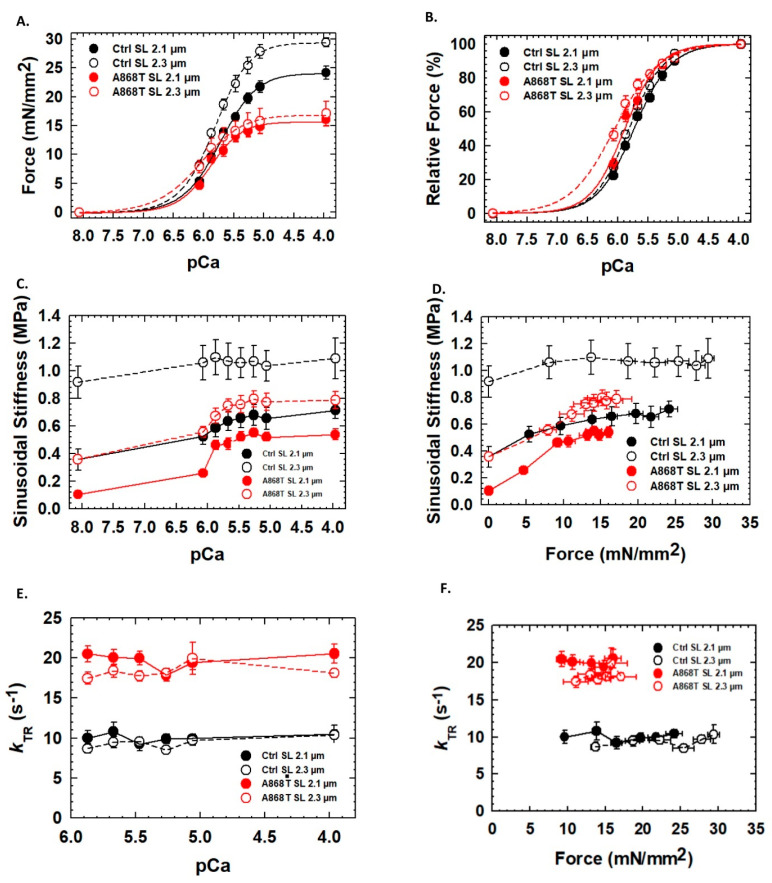
**Impacts of the A868T variant on the mechanics of cardiac muscle contraction.** (**A**,**B**) Isometric force and Ca^2+^ sensitivity: A868T CMPs presented a reduction in maximum steady-state and higher myofilament Ca^2+^ sensitivity. (**C**,**D**) Sinusoidal stiffness: A868T CMPs showed lower cross-bridge populations at all levels of activation. (**E**,**F**) Kinetics of tension redevelopment (*k_TR_*): A868T CMPs displayed faster *k_TR_* at all levels of calcium activation. Data are shown as mean ± S.D.; *n* = 4–5 per group (technical replicates).

To assess cardiac muscle contraction, we first evaluated the Ca^2+^ dependence of the steady-state isometric force of cardiac muscle preparations (CMPs). While the maximal force of activation was statistically significantly elevated in control CMPs upon stretch (i.e., 2.3 vs. 2.1 µm SL), it was not statistically different in A868T CMPs (Table 1). CMPs containing the A868T variant showed a statistically significant reduction in maximum steady-state isometric force compared to the control CMPs (Figure 3A and Table 1) at both SLs tested. Both control and A868T CMPs also show a statistically significant higher Ca^2+^ sensitivity of force upon stretch, implying that myofilament length-dependent activation is preserved in both tissues (Table 1). Moreover, A868T CMPs displayed a statistically significant higher myofilament Ca^2+^ sensitivity of force compared to control CMPs at both SLs tested (Figure 3B and Table 1) [43,44]. Hill coefficients (n_Hill_) were not statistically different within and between groups, suggesting no changes in thin filament cooperative activation upon stretch and between control vs. A868T CMPs [43]. Sinusoidal stiffness (SS) was used to evaluate the overall number of cross-bridges, with A868T CMPs displaying a lower SS at all levels of calcium activation (Red line, Figure 3C, Table 1) compared to the control CMPs (Black line, Figure 3C, Table 1). SS was also significantly lower at all levels of force activation (Figure 3D) in A868T CMPs when compared to the control CMPs. The kinetics of tension redevelopment (*k*_TR_) allows measurement of the kinetics of myosin reattachment to actin at different levels of Ca^2+^ activation after the isometric force has reached a steady state. Such measurements allow us to assess the dynamics of thin filament regulatory units when performed at submaximal Ca^2+^-activation levels. Maximum *k*_TR_ values were statistically significantly faster in A868T CMPs compared to control CMPs (Table 1), with A868T CMPs exhibiting faster *k*_TR_ at all levels of calcium activation compared to the control CMPs (Figure 3E). When *k*_TR_ was plotted versus isometric force, A868T CMPs also showed faster *k*_TR_ values compared to control CMPs at all force levels (Figure 3F). Mathematical modeling predicts that the rate of cross-bridge detachment (*g*) is faster in A868T CMPs with a slightly faster attachment rate (*f*) (Figure S3 and Table S1) [43,44]. Moreover, the assumption that A868T CMPs have a faster detachment rate (*g*) supports the notion that the reduced force generated by A868T CMPs is due to abnormalities in cross-bridge interactions. Together, the muscle mechanics data and the mathematical modeling suggest that the changes in the kinetics of A868T cardiac muscle are not solely due to an elevated stiffness in the muscle but also due to changes in the sarcomere structure that alter the interaction of thin and thick filaments, via our proposed CP1 and CP2 two-pathway mechanism, ultimately leading to disease.

### 2.4. Cardiac Muscle Preparations Obtained from the Patient’s Heart Have Larger Inter-Filament Lattice Spacing

**Figure 4:**
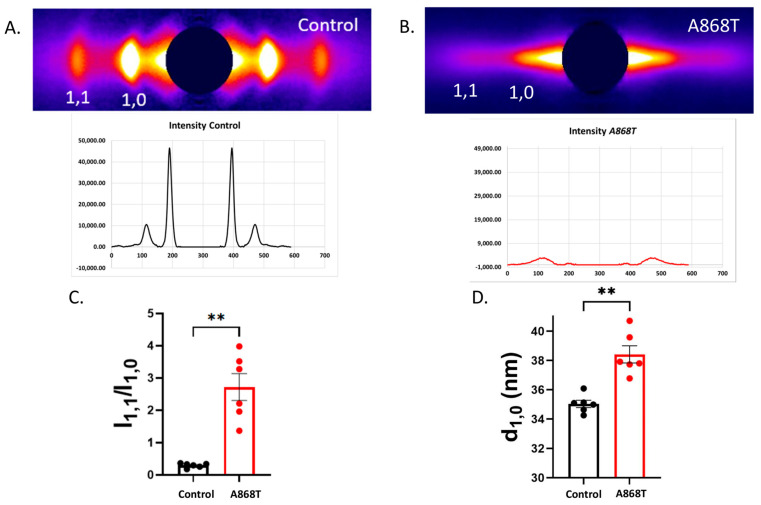
**Small-angle equatorial X-ray diffraction patterns from A868T cardiac tissue**. (**A**,**B**) Representative images of small angle equatorial X-ray diffraction patterns from control and A868T cardiac tissue. Histogram plots demonstrate the intensity of the equatorial reflections in both tissues. Overall, a reduction i intensity of the 1,0 and 1,1 reflections was observed in *A868T* variant cardiac tissue. (**C**) I_1,1_/I_1,0_ is increased in *ACNT2* A868T variant cardiac tissue as compared to WT. (**D**) Interfilament lattice spacing, d_10_, is elevated in A868T variant cardiac tissue. Data are shown as mean ± S.E; *n* = 6 per group (technical replicates), ** *p* < 0.01.

Small-angle X-ray fiber diffraction of muscle is the method of choice for investigating sarcomere structure, including the interaction of the myosin heads and actin during cross-bridge cycling [45]. Equatorial X-ray diffraction patterns from the control myocardium (Figure 4A) were compared to those from the A868T variant myocardium (Figure 4B). The equatorial X-ray diffraction pattern arises from the hexagonal packing of the thick and thin filaments in the sarcomeres. The two strongest reflections in the equatorial pattern are the 1,0 and 1,1 reflections [45,46,47,48]. The 1,0 and 1,1 diffraction peaks are clear and sharp in the control myocardium and weak and diffuse in the A868T variant myocardium, indicating a substantial degree of disorder in the sarcomeres of the mutant myocardium. The ratio of the intensities of the 1,1 equatorial reflection to that of the 1,0 reflection (I_1,1_/I_1,0_) is an indicator of the relative degree of association of myosin heads with actin [49,50] and is substantially greater in A868T than in control (Figure 4C), suggesting an elevation in the numbers of myosin heads shifting away from the thick filament backbone towards the actin filaments. An increase in I_1,1_/I_1,0_, however, does not necessarily imply elevated stereo-specific actin binding, which appears to be reduced, as indicated by a lower SS [49]. The separation of the 1,0 reflections allows calculation of the interfilament lattice spacing (LS), d_1,0_, related to the distance between thick and thin filaments [50,51]. LS in A868T variant CMPs is significantly larger (by ~10%) than in control samples. Larger lattice spacing results in myosin heads cycling more rapidly, as suggested by the *k*_TR_ data (Figure 3E,F). Wider lattice spacing can be explained by our proposed CP2 pathway, whereby altered interactions between α-actinin 2 and titin lead to changes in lattice spacings. In turn, increased I_1,1_/I_1,0_ can be explained by alterations in titin interactions with myosin, resulting in elevated OFF to ON transitions in the myosin heads [52]. Alterations in LS have also been shown to be associated with impaired force production and has been associated with cardiac disease [51]. Lastly, we analyzed the angular width of the X-ray reflections (angle sigma) from the X-ray diffraction experiments as a measure of the angular disarray of the myofibrils. The angle sigma measured in our A868T variant CMPs was 0.188 ± 0.32 rad (*n* = 8) as compared to 0.191 ± 0.011 rad from the control (*n* = 8). The radial width of the reflections (width sigma) can be used as a measure of the degree of LS inhomogeneity. Width sigma was 3.93 ± 0.8 [10^−3^ nm^−1^] (*n* = 8) in the A868T variant as compared to 4.13 ± 0.55 [10^−3^ nm^−1^] in control (*n* = 8). Both measurements were not significantly different, implying that the degree of myofibrillar angular disarray or LS inhomogeneity did not differ between the two types of muscle (Figure S4) [45].

### 2.5. Molecular Interactions of ACTN2 *A868T* Variant with Surrounding Sarcomere Proteins

To further investigate the underlying molecular dynamics (MD) interactions of EF 3–4 with α-actinin 2 or titin, we performed MD simulations of EF 3–4 binding to the α-actinin 2 neck region or titin Z-repeat 7 peptides. We first present in Figure S5 root mean square deviations (RMSDs) of the entire protein backbone; these data reflect how much the protein conformation changed from the input structure and whether the final conformations continue to change during the simulation. Figure S5 demonstrates that the system relaxes to a set of conformations that appear to be stable for the duration of the simulations. We also conducted analyses of the root mean square fluctuations of the protein backbone to assess the protein’s mobility (Figure S6). Our analyses revealed that a loop region near the mutation site of the α-actinin system showed higher fluctuations (Figure S6), indicating increased flexibility with the mutation. To assess whether this flexibility of the loop region would impact EF 3–4 binding to the actinin neck or titin peptide, we computed bindingfree energies using the MM/GBSA method (see extended methods for further details). Overall, the titin systems exhibited free energies of 15 kcal mol^−1^, which were more negative than the actinin systems, indicating stronger binding of the titin peptide to EF 3–4 (Figure S7). No significant difference, however, was noted between WT and A868T, suggesting that the mutation did not impact peptide binding. We next plotted a map of residues bound via hydrogen bonding (Figure S8) that depicts interactions between EF 3–4 and the bound peptides. While the actinin and titin systems each present unique peptide binding patterns, WT and A868T share almost the same patterns, suggesting that the binding of peptides was not perturbed due to this variant.

**Figure 5:**
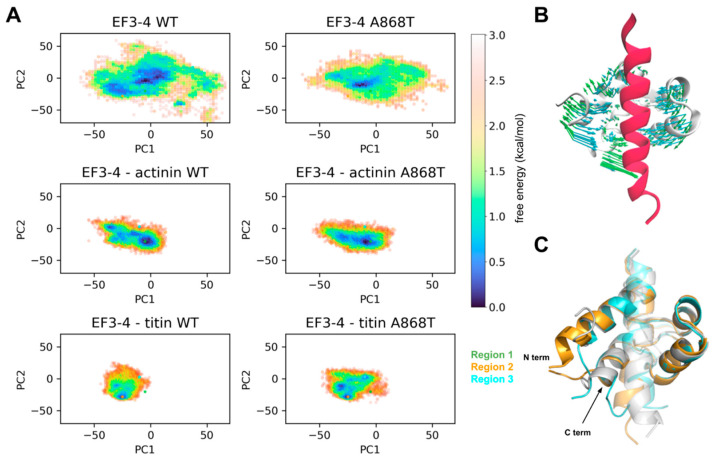
**Principal component analysis**. (**A**) Free energy landscapes along PC1 and PC2 of WT (left column) and A868T (right column) of the three systems. Free energies were converted from the probability density distributions using G = −k_B_TP, where k_B_ is Boltzmann’s constant, T is temperature (310 K), and P is the probability density. Low energy areas are color-coded and indicated by asterisks. (**B**) PC1 (cyan arrows) and PC2 (green arrows) projected onto the EF 3–4–actinin complex. The bound peptide is colored red. (**C**) Superposition of structures of EF 3–4 WT–actinin from region 1 (white), EF 3–4 WT–titin from region 2 (orange), and EF 3–4 A868T–titin from region 3 (cyan).

Finally, to examine structural changes involving the entire protein, we performed principal component analysis (PCA). For this analysis, we used the atomistic Cartesian coordinates for each snapshot of the EF 3–4 WT trajectories (i.e., time-dependent changes in the protein structure) to construct the PC bases, onto which trajectories from other systems were projected. This allowed us to determine large-scale conformation changes that occurred during the simulation. We then constructed free-energy landscapes by plotting the distributions along the first two principal components (PC1 and PC2), which allowed us to identify high-probability and, therefore, thermodynamically favorable, low-energy areas (Figure 5A, asterisks). In other words, these low-energy areas are indicative of conformations sampled more frequently during the simulations. Firstly, we examined the structural motions described by PC1 and PC2 by projecting these two components to the EF 3–4 structure. As shown in Figure 5B, PC1 (cyan arrows) captures the “clamping” of the EF 3–4 to bind the peptide, while PC2 (green arrows) represents the “twisting” motions of the EF 3–4. Notably, compared to the broad distribution of EF 3–4 alone, both peptide-bound systems present more constricted conformational distributions, indicating that the peptide binding stabilizes the clamped EF 3–4 conformation. The free energy landscapes of the WT and A868T actinin-bound systems are very similar, indicating no significant changes in the conformational distribution upon mutation. Intriguingly, while WT condenses to one low-energy area (orange asterisk), in the titin-bound system, A868T exhibits more diverse conformations, indicated by the multiple low-energy areas, which we speculate might compete with the native binding mode exhibited for the WT. We next compared the structures of WT in region 2 and A868T in region 3 (Figure 5C). Differences were noted at the N- and C-terminal. We attribute the N-terminal difference to terminal flexibility artifacts since the structure simulated in our study is a truncated form (N-terminal) of the full-length structure of α-actinin 2. The difference at the C-terminal, however, might provide structural insights, as the A868T C-terminal is shifted towards the bound peptide, causing the peptide to bend. Interestingly, the actinin neck region was also bent, which coincides with a higher binding free energy relative to the titin-bound system. Overall, the redistribution of the EF 3–4–titin conformation and multiple low energy areas with mutation indicate that there are non-productive titin-binding poses that could compete with the native titin-binding pose, which may impair actinin’s functional role in binding titin. Moreover, this flexibility at the C-terminus could also impact binding to other Z-disc proteins, such as PDLIM1, which is known to bind to α-actinin 2 C-terminal ESDL sequence [53].

## 3. Discussion

**Figure 6:**
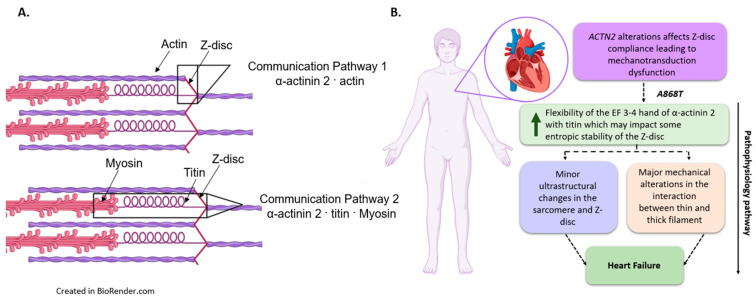
**Summary of proposed pathophysiological pathways.** (**A**) **Communication pathways.** Communication Pathway 1 (**CP1**) proposes a relationship between α-actinin 2 and actin that regulates thin filament activation. Communication Pathway 2 (CP2) proposes an interaction between α-actinin 2/titin/myosin that controls LS, thick filament activation, and cross-bridges kinetics. (**B**) **Model for α-actinin 2 variant *A868T* influences cardiac function, leading to pathology.** Summary of the ultrastructural and mechanical changes caused by the A868T variant on α-actinin 2 and the predicted role in the Z-disc of the sarcomere.

Our study demonstrates that a previously described α-actinin 2 variant with uncertain significance is likely to be involved in the etiology of cardiac disease in humans. It also establishes the importance of the EF 3–4 domain of α-actinin 2 for the proper function of the Z-disc with subsequent disruption of actomyosin interactions in the sarcomere [54]. Although α-actinin 2 EF-hand 3–4 region is highly conserved among species, the particular residue where the variant is located is not as highly conserved (Figure A1). While many species have alanine (A) at the location of A868 in human α-actinin 2, substitutions by proline (P) and glutamine (Q) were identified in some species (Figure A1). Interestingly, through comparative sequence alignment, we found four small mammal species that have threonine (T) in the amino acid position equivalent to human 868, as in our A868T variant patient samples (Figure A2). This finding suggests that the presence of a hydrophobic residue at this position is crucial for the EF-hand 3–4 domain in humans. In addition, our results with CMPs suggest evidence for two communication pathways: the CP1 pathway, from the Z-disc directly to the thin filaments, and the CP2 pathway, from the Z-disc directly to titin and indirectly to the thick filaments. In this framework, the elevated myofilament Ca^2+^ sensitivity can be explained by CP1, while elevated LS and I_1,1_/I_1,0_ can be explained by CP2 (Figure 6A).

### 3.1. Sarcomere-Based Ultrastructural and Functional Changes Are Observed in the *A868T* Variant

In this study, we have presented compelling evidence for the impact of the A868T variant on cardiac muscle performance. Variants in the *ACTN2* gene are associated with abnormal myofilament arrays with focal loss of myofibrils, which aligns with similar findings in *C. elegans* and *Drosophila* models containing *ACTN2* variants [54,55]. These findings suggest a compensatory mechanism whereby other binding proteins, such as actin, may adjust their function to maintain sarcomere architecture [54]. In this study, we showed that the A868T variant leads to higher flexibility of the α-actinin 2 EF 3–4 hand, potentially affecting the entropic stability of the Z-disc. Changes in Z-disc structural arrangements may act as compensatory mechanisms to ensure the integrity of protein–protein interactions. It has been reported that a less compact Z-disc structure results in abnormal Z-disc function, subsequently impacting cardiac contraction and signaling [56]. Notably, even though both sarcomere structural and functional changes were evident in the cardiac biopsies carrying the A868T variant, no major structural changes directly in the Z-disc were observed. It is possible that high-resolution cryo-electron microscopy studies could reveal subtle structural abnormalities, which cannot be achieved with conventional transmission EM.

Here, we showed that A868T CMPs exhibited a significantly lower maximal contractile force, shorter cross-bridge attachment time, and faster cross-bridge kinetics (Figure 3). It has been demonstrated that myofilament LS influences the kinetics of muscle contraction (i.e., larger LS equals faster muscle kinetics and vice versa) [44,46,57,58]. Based on this knowledge, we propose that a properly structured Z-disc plays a crucial role in preserving the optimal distance between thick and thin filaments, as reflected in the interfilament LS, for cross-bridge cycling and force generation [59]. Even a slight disruption in Z-disc stability, such as higher flexibility of the α-actinin EF 3–4 hand, could lead to alterations in myofilament LS, ultimately affecting contractility.

Our hypothesis gains support from both functional data and X-ray diffraction findings, in which a significantly larger myofilament LS was observed in A868T CMPs when compared to the control (Figure 4). We believe that several of the changes in muscle kinetics observed with the A868T CMPs can be explained by wider LS: (1) wider LS has been shown to elevate *k_TR_* [44,46]; (2) wider LS will reduce the time myosin spends attached to actin (i.e., we observed faster detachment rate (Supplemental Table S1), which will lead to lower maximal force; (3) lower maximal force can be correlated with a smaller number in overall number of cross-bridges during contraction (i.e., lower sinusoidal stiffness). However, how can we reconcile reduced SS with elevated I_1,1_/I_1,0_? We believe this can be explained by alterations in the CP2 pathway. It is known that titin can modulate the thick filament OFF–ON transitions [52]. In this case, altered interaction between α-actinin 2 and titin could be triggering an increase in the myosin heads in the ON state, which can explain elevated I_1,1_/I_1,0_. Despite more myosin heads positioned away from the thick filament backbone and closer to thin filaments, as indicated by higher I_1,1_/I_1,0_, the heads are either weakly interacting with actin or generating less force, as suggested by lower SS and lower maximal force. In summary, our study highlights that even minimal alterations in the structure and function of α-actinin 2, as seen with the A868T variant, can result in cardiac contractile dysfunction. By impacting the spatial organization of thick and thin filaments within the sarcomere, the A868T variant disrupts the delicate balance necessary for proper cardiac muscle contraction.

### 3.2. Interactions of Proteins Associated with the Z-Disc in the *A868T* Variant

There are several proteins surrounding the Z-disc that could affect the kinetics of muscle contraction. Gregorich et al. propose the enigma homologue protein (ENH) located in the Z-disc as one protein with a direct impact on muscle kinetics. Ablation of ENH protein leads to muscle dysfunction and the development of dilated cardiomyopathy. It appears that ENH, which is part of the PDZ/LIM family of proteins, disturbs actomyosin cross-bridge interactions by inducing structural changes in the Z-disc that affects the alignment of actin and myosin filaments similarly to our proposed CP1 and CP2. Our simulation data indicate that the A868T variant alters the interaction between the mutated protein and titin, which was evidenced by differences in the complex’s distribution as assessed by PC analysis (Figure 5). Thus, the CP2 pathway might be affected by the A868T variant where the increase in α-actinin 2 flexibility yields changes in titin function, consequently increasing thick filament OFF-ON transitions, as indicated by the observed higher I_1,1_/I_1,0_. As discussed above, this higher I_1,1_/I_1,0_ does not necessarily imply more actin binding by cross-bridges. Further studies are warranted to evaluate whether similar perturbations may occur on other abundant sarcomere proteins, such as paladin (PALLD), synaptopodin (SYNPO2), myozenin (MYOZENIN), α-actinin 1 (ACTN1), gap junction protein α 1 (GJA1) and ENH proteins [16,60] and potentially their intrinsically disordered regions important to sarcomere function [61]. In any event, the A868T variant adopted a partially opened complex that was less capable of binding titin, which could potentially impact the Z-disc functionality despite preserving its actin-binding properties. Hence, it is likely that the A868T variant’s interaction with other sarcomere proteins influences Z-disc behavior, in addition to its interactions with actin.

### 3.3. Can Changes in Titin Flexibility Alter the Kinetics of Cardiac Muscle?

Titin plays an important role in myogenesis in the assembly of the Z-disc and interaction with different components of the thick filament. It appears that the stability of the CP2 pathway directly impacts the mechanical strength of the sarcomere. There are specific Z-repeats of titin that interact with the Spectrin Repeats (SR) of α-actinin 2. Our MD simulations suggest that the A868T variant affects interactions between these sarcomere proteins that subsequently affect the kinetics of the contraction of the sarcomere, supporting our main hypothesis. Previous authors have reported that titin Z-repeats and their distributions might vary based on the type of striated muscle, and this could be the reason why our patient did not report any other muscular issues besides those cardiac associated [62,63]. It seems that overexpression of the Z1 and Z2 repeats of titin and T-cap can directly affect the assembly of the Z-disc. Although we were not able to elucidate the specific Z-repeat that might be affected by the A868T variant, it is important to highlight that variants in the Z-repeats of titin and α-actinin 2 can impact the dynamics of other sarcomere proteins [62,63]. Equally important is titin’s direct impact on the sarcomere’s ultrastructural properties. Here, we observed some minimal changes at the ultrastructural level of the sarcomere, including shorter SL and reduced myofibrillar width. Titin has been proposed to act as an LS regulator with subsequent effects on the mechanics of the sarcomere. Similar findings have been described in previous studies where abnormal titin interactions in the Z-disc affect sarcomere measurements such as Z-disc circumference, myofibrillar area, and surrounding protein expression in striated muscle [63]; although, these findings can vary depending on the type of muscle and titin isoforms. Our MD simulations suggest that the A868T variant alters the interaction between titin and α-actinin 2, and with wider LS observed in our X-ray diffraction experiments, we propose that the changes in muscle mechanics we observe can be attributed to alterations of sarcomere architecture [64]. Nonetheless, the importance of titin at the functional level of the sarcomere is well known and, thus, something that could be impacted by the A868T variant samples [63].

### 3.4. Implications of the *A868T* Variant as a Contributor to the Development of Cardiac Disease

We sought to identify a possible mechanism by which the A868T variant might lead to disease. Our results strongly suggest that the A868T variant alters CP1 and/or CP2 as a pathophysiological mechanism. The MD simulations data suggest that the abnormal interaction between α-actinin 2 and titin may nucleate a maladaptive change in the sarcomere. Although no major alterations between the interaction of α-actinin 2 and actin have been identified, it is important to point out that the elevation in Ca^2+^ sensitivity observed in Figure 3B might result from conformational changes in the thin filament due to the variant via our proposed CP1 (Figure 6A). Therefore, our data provide new insights into the Z-disc physiology and, consequently, how alterations in thin- and thick-filament function via α-actinin 2, directly or indirectly, can lead to alterations in the contractile properties of the sarcomere. Additional studies using cryo-electron microscopy, as have been conducted with isolated cardiac thin filaments [65,66], could allow a deeper structural understanding of how Z-disc proteins communicate with thin and thick filament proteins to modulate contraction.

## 4. Materials and Methods

### 4.1. Human Heart Muscle Samples

The explanted control donor human heart used for our control samples was obtained from the Vanderbilt University Medical Center. Control samples were taken from a de-identified 44-year-old female normal donor whose heart was procured but not transplanted. The cause of death for the donor is not available but it was not due to cardiac-related issues, which allowed for inclusion into the Vanderbilt Heart Failure Biorepository (VUMC IRB #131978 and #202301). The control data reported in this manuscript were obtained from this single donor heart; therefore, the data points all represent technical replicates. The de-identified α-actinin A868T heart sample was obtained from the Vanderbilt University Medical Center after the patient’s informed consent was obtained (VUMC IRB #131978 and #202301). The α-actinin A868T variant data reported in this manuscript were obtained from this single patient heart; therefore, the data points represent technical replicates.

### 4.2. Electron Microscopy

Samples from the proband explanted heart were fixed in 3% buffered glutaraldehyde at the time of transplant, processed into Spurr resin, and thin sections were cut for transmission electron microscopy per the standard protocol in the VUMC Clinical Pathology lab. Normal controls were identified from previously stored endomyocardial biopsies. Thin sections were imaged with an FEI Tecnai 12 transmission electron microscope. Five micrographs at 7100× and 14,000× were reviewed by clinical pathologists and used for analysis. Sarcomere length and myofibril width from EM images were quantified using ImageJ and GraphPad software version 9.4.1.

### 4.3. Immunofluorescence

Histological preparation slices of patient myocardium were embedded in optimal cutting temperature compound (OCT), frozen at −80 °C, and cut using microtome equipment. Immunostaining was carried out using prepared tissue slides fixed in ice-cold fixation buffer (4% paraformaldehyde) for 20 min at room temperature. The fixed tissue was washed 3× for 10 min in Permeabilization Buffer (10% FBS, 0.2% Triton X-100 in PBS) and then incubated with Collagenase Permeabilization Buffer (Collagenase II and IV in permeabilization buffer) for 30 min. Immunostaining was followed by incubating the primary antibody for α-Actinin 2 (GeneTex #GTX103219) or Titin (Novus #NBP1-88071) overnight at 4 °C. This was followed by 3× washes of Permeabilization Buffer for 5 min and incubation of secondary antibody (Alexa Fluor, Invitrogen #A-21206) for 2 h at room temperature. After the removal of the secondary antibody, 3× washes of Permeabilization Buffer for 5 min were performed, followed by incubation of DAPI (concentration 1:5000) for 5 min. The final 3× washes of Permeabilization Buffer were performed to clean the slides. Mounted slides were imaged using Carl Zeiss LSM 880 Confocal Microscope Systems.

Fluorescence Intensity was measured using ImageJ software. Each image was separated into different channels (blue channel for DAPI dye and green and red channels for α-actinin 2 and titin antibodies, respectively). Images with actinin 2 or titin antibody were selected, and an area within the image was measured and analyzed. The areas containing actinin 2 striations in Figure 2C–F were selected, and mean fluorescence intensity along with background fluorescence was measured for analysis. The background mean was subtracted from the fluorescence mean to provided an accurate value for the fluorescence of the area. This process was repeated 3× per image on different locations of the images selected for fluorescence analysis Figures S1 and S2.

Z-bodies and titin spot counts were measured using ImageJ software. In Figure 2C,D, tissue slides were labeled with α-actinin 2 antibody and an individual count of each Z-body was made. This process was repeated 3× per image (Figure S1). A similar process was completed in Figure 2E,F, where we present an image labeled with titin. This process was conducted in triplicate per image with the same selected area (Figure S2). The average numbers of Z-bodies and titin spots were plotted and analyzed using GraphPad software version 9.4.1, as presented in Figure 2M,N.

### 4.4. Cardiac Muscle Preparations (CMPs)

CMPs were isolated from patients’ heart samples and prepared according to established protocols [44]. CMPs were skinned using 1% Triton X-100 for four hours at 4 °C. CMPs were mounted to a force transducer (model 403A, Aurora Scientific Inc., Ontario, CA, USA), and length was controlled with a high-speed servomotor (Aurora Scientific Inc. Model 312C). The steady-state isometric Ca^2+^-dependent force was measured by exposing the CMPs to a series of solutions ranging from low Ca^2+^ concentration pCa 8 (10^−8^ M free Ca^2+^) to high Ca^2+^ concentrations pCa 4 (10^−4^ M free Ca^2+^) at ~30 °C [67]. Sarcomere length was set to 2.1 μm using HeNe laser diffraction (at pCa 8); CMPs were then incubated in pCa8 with 3% dextran T-500 solution for 1 h before the experiments [44]. The use of dextran reduces the myofilament lattice spacing to close to the physiological value [44]. Data were fitted using a two- or three-parameter sigmoidal Hill equation, as previously described [43,44,68,69].

### 4.5. Muscle Mechanics

The rate of tension redevelopment (*k*_TR_) was measured after force levels reached a steady state at each level of Ca^2+^ activation. *k*_TR_ was obtained by shortening the CMPs to 20% of its original length (L_0_), followed by a 25% rapid restretch, and shortening back to L_0_ [43,44,70]. *k*_TR_ was calculated from individual measurements as previously described [44].

Sinusoidal stiffness (SS) was obtained after the force reached a steady state in each pCa solution at ~30 °C by measuring the changes in SL and their respective changes in force. CMPs oscillated at a small length perturbation of 0.2% peak-to-peak of the initial length at a frequency of 100 Hz with a sampling rate of 1 kHz. The SS data were analyzed with R studio Fast Fourier transform, and the values were reported in megapascals. The data were fitted using a four-parameter sigmoidal Hill equation, as previously described [43,44].

### 4.6. X-ray Diffraction

*Muscle preparations:* The muscle tissue was permeabilized as previously described [45]. Briefly, the muscle was permeabilized in skinning solution (2.25 mM Na_2_ATP, 3.56 mM MgCl_2_, 7 mM EGTA, 15 mM sodium phosphocreatine, 91.2 mM potassium propionate, 20 mM imidazole, 0.165 mM CaCl_2_, 15 mM 2,3-butanedione 2-monoxime (BDM), 1% Triton X-100 and protease inhibitor cocktail (complete, Sigma Aldrich, St. Louis, MO, USA)) for ~30 min before being split into smaller CMPs. The CMPs were transferred into fresh skinning solutions and incubated overnight at 4 °C. The CMPs were washed with fresh, relaxing solution (pCa 8: 2.25 mM Na_2_ATP, 3.56 mM MgCl_2_, 7 mM EGTA, 15 mM sodium phosphocreatine, 91.2 mM potassium propionate, 20 mM imidazole, 0.165 mM CaCl_2_) 3× for 10 min each to wash out BDM and Triton X-100. Muscles were further dissected into strips, clipped with aluminum T-clips, and stored in a cold, relaxing solution for the day’s experiments.

### 4.7. Small-Angle X-Diffraction

Equatorial X-ray diffraction patterns were collected from freshly permeabilized muscle strips using the small-angle instrument on the BioCAT beamline 18ID at the Advanced Photon Source, Argonne National Laboratory, as described in [71]. Diffraction patterns were collected at a SL of 2.3 μm with a 1 s exposure time at an incident flux of ∼3 × 10^12^ photons per second on a CCD-based X-ray detector (Mar 165; Rayonix Inc. Evanston, IL, USA). The data were analyzed using the “equator” data reduction routine from the MuscleX V1.22.0 software package developed by BioCAT [72], as described in [49].

### 4.8. MD Simulations and Analysis

To study the molecular basis of EF 3–4 interacting with α-actinin or titin, we performed molecular dynamics (MD) simulations of the following systems: (1) EF 3–3, (2) EF 3–4 with the α-actinin neck region bound, and (3) EF 3–4 with the equivalent region of titin, Z-repeat 7, bound (PDB: 1H8B) [73]. The structure of EF 3–4 with the α-actinin neck region bound was constructed from the crystal structure of α-actinin (PDB: 4D1E) [15]. In particular, since the PDB file contains coordinates of only one monomer, the dimer structure was generated using the sym operation in UCSF Chimera [74] (i.e., “sym biomt biomtSet”). From the dimeric structure, the region corresponding to the EF 3–4 with titin was then extracted and used as the starting structure of the EF 3–4 with the α-actinin neck region. In addition, wild-type and A868T mutations were simulated for each system. Therefore, a total of six cases were simulated.

MD simulations were performed following a similar protocol as previously reported [75]. Specifically, the AMBER ff14SB [76] force field was used for protein atom parameterization. Each system was solvated in a TIP3P [77] water box with the distance between protein to water box wall set to 12 Å. 0.15 M K^+^ and Cl^−^ ions were added to mimic the ionic strength encountered in a physiological environment. Energy minimization was next performed with a non-bonded interactions cutoff value of 10 Å, a 2fs time-step, with SHAKE [78] constraints applied to the bonds involving hydrogen atoms, and a restraint of 10 kcal mol^−1^ Å^−2^ imposed on the protein backbone. Each system was then heated from 0 to 300 K in two heating stages. During the first stage, the system was heated from 0 to 300 K under an NVT ensemble with a restraint of 10 kcal mol^−1^ Å^−2^ imposed on the protein backbone to immobilize these atoms. In the next heating stage, the system was heated from 0 to 300 K under an NPT ensemble with a reduced restraint of 3 kcal mol^−1^ Å^−2^ imposed on the protein backbone to allow for slight relaxation. The system was then subject to a 1 ns equilibration at 300 K with a further reduced restraint of 1 kcal mol^−1^ Å^−2^ imposed on the protein backbone. A Langevin thermostat with a collision frequency of 3 ps^−1^ was used during the simulation. Finally, starting from the equilibrated configuration, 1 μs production MD with no restraints was performed in triplicate with randomized initial velocities.

Clustering analysis, root mean square deviation (RMSD)/root mean square fluctuation (RMSF) calculations, hydrogen bonds, and principal component analysis (PCA) were performed using CPPTRAJ [79]. To estimate the binding free energies between EF 3–4 and α-actinin or titin, MM/GBSA calculations were performed using down-sampled production MD trajectories with a salt concentration of 0.15 M, a surface tension of 0.0072 kcal mol^−1^ Å^−2^ (default value) and a Generalized Born method (igb = 5) via MMPBSA.py [80].

### 4.9. Statistical Analysis

Data were reported as mean ± S.D. or mean ± S.E. For electron microscopy, immunofluorescence, and X-ray diffraction experiments, statistical analyses between the two different groups were performed using an unpaired Student’s *t*-test with GraphPad (version 9.4.1 for Windows, GraphPad Software, San Diego, CA, USA. * *p* < 0.05 and ** *p* < 0.01 were considered statistically significant. For the muscle mechanics experiments, statistical analyses within and between groups were performed using the One-Way ANOVA Student–Newman–Keuls Method with SigmaPlot v12 (Systat Software, Inc., San Jose, CA, USA), where statistical significance was shown as * *p* < 0.05 and ** *p* < 0.01 for comparisons within the same group, and # *p* < 0.05, ## *p* < 0.01, and ### *p* < 0.001 for comparisons between groups.

## 5. Conclusions

Our study identifies A868T as a possible pathogenic variant of *ACTN2* in cardiac muscle that leads to severe ventricular fibrillation and an HCM-like phenotype with diastolic dysfunction (Figure 6B). Overall, cardiac muscle mechanics were compromised, suggesting abnormal actomyosin interactions due to alterations in structural interactions between α-actinin 2, titin (CP2), and actin (CP1) within the Z-disc. Our modeling data predict differences in the interactions between α-actinin 2 and titin. Our muscle mechanics and small-angle X-ray diffraction studies suggest that there is a wider LS between the thin and thick filaments accompanied by an elevation in cross-bridge cycling but with a shorter attachment time. The wider LS might be related to alterations in the interactions between the variant and titin, affecting the CP2 pathway and leading to the functional changes that were observed in this study. Overall, the data suggest that the A868T variant alters the mechano-sensing role of α-actinin 2 and disrupts the functional performance of the sarcomere, ultimately leading to cardiac disease (Figure 6B).

### Limitations

A 134 gene cardiomyopathy GeneDx panel, rather than full genome sequencing, was performed during the patient’s clinical assessment. This panel covers the most relevant pathological genes. We are aware that a combination of mutated genes and environmental conditions might both be involved in the etiology of the patient’s cardiac disease; however, a more detailed genetic study was not possible due to patients’ families declining follow-ups in the medical genetics clinic; therefore, better genetic study is not possible at the moment. Future studies with iPSC-cardiomyocytes and/or animal models are required to clarify the role of the α-actinin A868T variant in the development of cardiomyopathy in humans.

## Figures and Tables

**Table 1:** **Muscle mechanics analysis of cardiac muscle preparations.** F_max_ = maximal steady-state isometric force. F_pass_ = force measured at low levels of Ca^2+^ (pCa 8). F*p*Ca_50_ = pCa needed to reach 50% of the maximal steady-state isometric force. F*n*_Hill_ = Hill coefficient, interpreted in terms of cooperative Ca^2+^-activation of the myofilaments (it was calculated from the pCa vs. force measurements). *k*_TRmax_ = rate of tension redevelopment measured after CMPs have reached maximal steady-state isometric force. SS_pass_ = sinusoidal stiffness measured at low levels of Ca^2+^ (pCa 8). SS_max_ = sinusoidal stiffness measured after CMPs have reached maximal steady-state isometric force. CMP = cardiac muscle preparations. One-way ANOVA, with post hoc Student–Newman–Keuls method, was used for statistical analyses. * *p* < 0.05, ** *p* < 0.01 (comparisons within the same group, i.e., Ctrl 2.1 µm vs. Ctrl 2.3 µm and A868T 2.1 µm vs. A868T 2.3 µm) ^#^ *p* < 0.05, ^##^ *p* < 0.01, ^###^ *p* < 0.001 (comparisons between different groups, i.e., Ctrl 2.1 µm vs. A868T 2.1 µm and Ctrl 2.3 µm vs. A868T 2.3 µm). Data are shown as mean ± S.D. *n* = 4–5 per group (technical replicates).

	Control (Ctrl)		α-actinin 2 Mutant (A868T)	
	Ctrl 2.1 µm	Ctrl 2.3 µm	A868T 2.1 µm	A868T 2.3 µm
**F_max (mN/mm_^2^_)_**	24.16 ± 1.13	29.38 ± 0.80 *	16.02 ± 1.13 ^#^	17.08 ± 2.06 ^#^
**F_pass (mN/mm_^2^_)_**	1.57 ± 0.31	2.94 ± 0.47 **	0.78 ± 0.32 ^#^	0.91 ± 0.11 ^###^
**F*p*Ca_50_**	5.72 ± 0.01	5.81 ± 0.02 *	5.88 ± 0.03 ^##^	6.06 ± 0.05 **^,###^
**F*n*_Hill_**	1.43 ± 0.06	1.54 ± 0.13	1.56 ± 0.06	1.22 ± 0.08
***k*** **_TRmax_** **(s^−1^)**	10.47 ± 0.55	10.37 ± 1.25	20.54 ± 1.22 ^#^	18.10 ± 0.51 ^#^
**SS_pass_ (MPa)**	0.36 ± 0.08	0.91 ± 0.12 *	0.10 ± 0.01 ^#^	0.35 ± 0.01 *^,#^
**SS_max_ (MPa)**	0.71 ± 0.06	1.09 ± 0.14 *	0.54 ± 0.04	0.79 ± 0.06 ^#^

## Data Availability

All code written in support of this publication is publicly available at https://github.com/huskeypm/pkh-lab-analyses (accessed on 9 September 2022). M.D. trajectories are available upon request.

## Supplementary Materials

The supporting information can be downloaded at: https://www.mdpi.com/article/10.3390/ijms241914572/s1.

## Appendix A

Evolutionary analysis of α-actinin 2 around EF 3–4 sequence reveals ~95% of conservation across 25 different species (Figure A1). Despite this high degree of conservation, substitutions of A868 were identified by proline (P) or, in one case, by glutamine (Q) (Figure A1). Figure A2 highlights four small mammal species (lesser Madagascar hedgehog, meerkat, gray mouse lemur, and ring-tailed lemur) that have a threonine (T) residue where the A868T variant is located in human α-actinin 2. It would be of great interest to know how these small mammals are adapted to living with a variant that is detrimental in human patients.

## References

[1] Sjoblom B., Salmazo A., Djinovic-Carugo K. (2008). Alpha-actinin structure and regulation. Cell. Mol. Life Sci..

[2] Taylor K.A., Taylor D.W., Schachat F. (2000). Isoforms of alpha-actinin from cardiac, smooth, and skeletal muscle form polar arrays of actin filaments. J. Cell Biol..

[3] Blanchard A., Ohanian V., Critchley D. (1989). The structure and function of alpha-actinin. J. Muscle Res. Cell Motil..

[4] Hampton C.M., Taylor D.W., Taylor K.A. (2007). Novel structures for alpha-actinin: F-actin interactions and their implications for actin-membrane attachment and tension sensing in the cytoskeleton. J. Mol. Biol..

[5] Masaki T., Endo M., Ebashi S. (1967). Localization of 6S component of a alpha-actinin at Z-band. J. Biochem..

[6] Squire J.M. (1997). Architecture and function in the muscle sarcomere. Curr. Opin. Struct. Biol..

[7] Mills M., Yang N., Weinberger R., Vander Woude D.L., Beggs A.H., Easteal S., North K. (2001). Differential expression of the actin-binding proteins, alpha-actinin-2 and -3, in different species: Implications for the evolution of functional redundancy. Hum. Mol. Genet..

[8] Gregorich Z.R., Patel J.R., Cai W., Lin Z., Heurer R., Fitzsimons D.P., Moss R.L., Ge Y. (2019). Deletion of Enigma Homologue from the Z-disc slows tension development kinetics in mouse myocardium. J. Gen. Physiol..

[9] Burnette D.T., Hayes J.B. (2023). The role of alpha actinin paralogs in cardiac hypertrophy and contractile force production. Biophys. J..

[10] Grum V.L., Li D., MacDonald R.I., Mondragon A. (1999). Structures of two repeats of spectrin suggest models of flexibility. Cell.

[11] Rusu M., Hu Z., Taylor K.A., Trinick J. (2017). Structure of isolated Z-disks from honeybee flight muscle. J. Muscle Res. Cell Motil..

[12] Grison M., Merkel U., Kostan J., Djinovic-Carugo K., Rief M. (2017). Alpha-Actinin/titin interaction: A dynamic and mechanically stable cluster of bonds in the muscle Z-disk. Proc. Natl. Acad. Sci. USA.

[13] Gautel M., Goulding D., Bullard B., Weber K., Furst D.O. (1996). The central Z-disk region of titin is assembled from a novel repeat in variable copy numbers. J. Cell Sci..

[14] Fukami K., Endo T., Imamura M., Takenawa T. (1994). Alpha-Actinin and vinculin are PIP2-binding proteins involved in signaling by tyrosine kinase. J. Biol. Chem..

[15] Ribeiro Ede A., Pinotsis N., Ghisleni A., Salmazo A., Konarev P.V., Kostan J., Sjoblom B., Schreiner C., Polyansky A.A., Gkougkoulia E.A. (2014). The structure and regulation of human muscle alpha-actinin. Cell.

[16] Lindholm M.E., Jimenez-Morales D., Zhu H., Seo K., Amar D., Zhao C., Raja A., Madhvani R., Abramowitz S., Espenel C. (2021). Mono- and Biallelic Protein-Truncating Variants in Alpha-Actinin 2 Cause Cardiomyopathy Through Distinct Mechanisms. Circ. Genom. Precis. Med..

[17] Chiu C., Bagnall R.D., Ingles J., Yeates L., Kennerson M., Donald J.A., Jormakka M., Lind J.M., Semsarian C. (2010). Mutations in alpha-actinin-2 cause hypertrophic cardiomyopathy: A genome-wide analysis. J. Am. Coll. Cardiol..

[18] Zech A.T.L., Prondzynski M., Singh S.R., Pietsch N., Orthey E., Alizoti E., Busch J., Madsen A., Behrens C.S., Meyer-Jens M. (2022). ACTN2 Mutant Causes Proteopathy in Human iPSC-Derived Cardiomyocytes. Cells.

[19] Broadway-Stringer S., Jiang H., Wadmore K., Hooper C., Douglas G., Steeples V., Azad A.J., Singer E., Reyat J.S., Galatik F. (2023). Insights into the Role of a Cardiomyopathy-Causing Genetic Variant in *ACTN2*. Cells..

[20] Kosaraju A., Goyal A., Grigorova Y., Makaryus A.N. (2022). Left Ventricular Ejection Fraction. StatPearls [Internet].

[21] Wu V.C., Takeuchi M. (2018). Echocardiographic assessment of right ventricular systolic function. Cardiovasc. Diagn. Ther..

[22] Kong W.K.F., Vollema E.M., Prevedello F., Perry R., Ng A.C.T., Poh K.K., Almeida A.G., Gonzalez A., Shen M., Yeo T.C. (2020). Prognostic implications of left ventricular global longitudinal strain in patients with bicuspid aortic valve disease and preserved left ventricular ejection fraction. Eur. Heart J. Cardiovasc. Imaging.

[23] Larson L.W., Gerbert D.A., Herman L.M., Leger M.M., McNellis R., O’Donoghue D.L., Ulshafer C., Van Dyke E.M., American College of Cardiology, American Heart Association (2006). ACC/AHA 2005 guideline update: Chronic heart failure in the adult. JAAPA.

[24] Reant P., Mirabel M., Lloyd G., Peyrou J., Lopez Ayala J.M., Dickie S., Bulluck H., Captur G., Rosmini S., Guttmann O. (2016). Global longitudinal strain is associated with heart failure outcomes in hypertrophic cardiomyopathy. Heart.

[25] Mignot A., Donal E., Zaroui A., Reant P., Salem A., Hamon C., Monzy S., Roudaut R., Habib G., Lafitte S. (2010). Global longitudinal strain as a major predictor of cardiac events in patients with depressed left ventricular function: A multicenter study. J. Am. Soc. Echocardiogr..

[26] Prondzynski M., Lemoine M.D., Zech A.T., Horvath A., Di Mauro V., Koivumaki J.T., Kresin N., Busch J., Krause T., Kramer E. (2019). Disease modeling of a mutation in alpha-actinin 2 guides clinical therapy in hypertrophic cardiomyopathy. EMBO Mol. Med..

[27] Saetersdal T.S., Myklebust R., Skagseth E., Engedal H. (1976). Ultrastructural studies on the growth of filaments and sarcomeres in mechanically overloaded human hearts. Virchows Arch. B Cell Pathol..

[28] Russell B., Curtis M.W., Koshman Y.E., Samarel A.M. (2010). Mechanical stress-induced sarcomere assembly for cardiac muscle growth in length and width. J. Mol. Cell Cardiol..

[29] Stein A.A., Thibodeau F., Stranahan A. (1962). Electron microscopic studies of human myocardium. JAMA.

[30] Solaro R.J., Leinwand L.A. (2012). Role of Sarcomeres in Cellular Tension, Shortening, and Signaling in Cardiac Muscle. Muscle Fundamental Biology and Mechanisms of Disease.

[31] Coscarella I.L., Landim-Vieira M., Rastegarpouyani H., Chase P.B., Irianto J., Pinto J.R. (2023). Nucleus Mechanosensing in Cardiomyocytes. Int. J. Mol. Sci..

[32] Pioner J.M., Racca A.W., Klaiman J.M., Yang K.C., Guan X., Pabon L., Muskheli V., Zaunbrecher R., Macadangdang J., Jeong M.Y. (2016). Isolation and Mechanical Measurements of Myofibrils from Human Induced Pluripotent Stem Cell-Derived Cardiomyocytes. Stem Cell Rep..

[33] Solaro R.J. (2016). Why we need to understand the mechanics of developing cardiac sarcomeres in humans. J. Physiol..

[34] Knoll R., Buyandelger B., Lab M. (2011). The sarcomeric Z-disc and Z-discopathies. J. Biomed. Biotechnol..

[35] Crocini C., Gotthardt M. (2021). Cardiac sarcomere mechanics in health and disease. Biophys. Rev..

[36] Chechenova M.B., Bryantsev A.L., Cripps R.M. (2013). The Drosophila Z-disc protein Z(210) is an adult muscle isoform of Zasp52, which is required for normal myofibril organization in indirect flight muscles. J. Biol. Chem..

[37] Cai L.X., Tanada Y., Bello G.D., Fleming J.C., Alkassis F.F., Ladd T., Golde T., Koh J., Chen S., Kasahara H. (2019). Cardiac MLC2 kinase is localized to the Z-disc and interacts with alpha-actinin2. Sci. Rep..

[38] Dabiri G.A., Turnacioglu K.K., Sanger J.M., Sanger J.W. (1997). Myofibrillogenesis visualized in living embryonic cardiomyocytes. Proc. Natl. Acad. Sci. USA.

[39] Sanger J.W., Chowrashi P., Shaner N.C., Spalthoff S., Wang J., Freeman N.L., Sanger J.M. (2002). Myofibrillogenesis in skeletal muscle cells. Clin. Orthop. Relat. Res..

[40] Gregorio C.C., Antin P.B. (2000). To the heart of myofibril assembly. Trends Cell Biol..

[41] van der Ven P.F., Schaart G., Croes H.J., Jap P.H., Ginsel L.A., Ramaekers F.C. (1993). Titin aggregates associated with intermediate filaments align along stress fiber-like structures during human skeletal muscle cell differentiation. J. Cell Sci..

[42] Tokuyasu K.T., Maher P.A. (1987). Immunocytochemical studies of cardiac myofibrillogenesis in early chick embryos. II. Generation of alpha-actinin dots within titin spots at the time of the first myofibril formation. J. Cell Biol..

[43] Landim-Vieira M., Johnston J.R., Ji W., Mis E.K., Tijerino J., Spencer-Manzon M., Jeffries L., Hall E.K., Panisello-Manterola D., Khokha M.K. (2019). Familial Dilated Cardiomyopathy Associated With a Novel Combination of Compound Heterozygous TNNC1 Variants. Front. Physiol..

[44] Gonzalez-Martinez D., Johnston J.R., Landim-Vieira M., Ma W., Antipova O., Awan O., Irving T.C., Bryant Chase P., Pinto J.R. (2018). Structural and functional impact of troponin C-mediated Ca(2+) sensitization on myofilament lattice spacing and cross-bridge mechanics in mouse cardiac muscle. J. Mol. Cell Cardiol..

[45] Ma W., Gong H., Jani V., Lee K.H., Landim-Vieira M., Papadaki M., Pinto J.R., Aslam M.I., Cammarato A., Irving T. (2022). Myofibril orientation as a metric for characterizing heart disease. Biophys. J..

[46] Landim-Vieira M., Ma W., Song T., Rastegarpouyani H., Gong H., Coscarella I.L., Bogaards S.J.P., Conijn S.P., Ottenheijm C.A.C., Hwang H.S. (2023). Cardiac troponin T N-domain variant destabilizes the actin interface resulting in disturbed myofilament function. Proc. Natl. Acad. Sci. USA.

[47] Song T., McNamara J.W., Ma W., Landim-Vieira M., Lee K.H., Martin L.A., Heiny J.A., Lorenz J.N., Craig R., Pinto J.R. (2021). Fast skeletal myosin-binding protein-C regulates fast skeletal muscle contraction. Proc. Natl. Acad. Sci. USA.

[48] van de Locht M., Donkervoort S., de Winter J.M., Conijn S., Begthel L., Kusters B., Mohassel P., Hu Y., Medne L., Quinn C. (2021). Pathogenic variants in TNNC2 cause congenital myopathy due to an impaired force response to calcium. J. Clin. Investig..

[49] Ma W., Gong H., Irving T. (2018). Myosin Head Configurations in Resting and Contracting Murine Skeletal Muscle. Int. J. Mol. Sci..

[50] Reconditi M. (2006). Recent Improvements in Small Angle X-ray Diffraction for the Study of Muscle Physiology. Rep. Prog. Phys..

[51] Squire J. (1981). The Structural Basis of Muscular Contraction.

[52] Ma W., Henze M., Anderson R.L., Gong H., Wong F.L., Del Rio C.L., Irving T. (2021). The Super-Relaxed State and Length Dependent Activation in Porcine Myocardium. Circ. Res..

[53] Elkins J.M., Gileadi C., Shrestha L., Phillips C., Wang J., Muniz J.R., Doyle D.A. (2010). Unusual binding interactions in PDZ domain crystal structures help explain binding mechanisms. Protein Sci..

[54] Moulder G.L., Cremona G.H., Duerr J., Stirman J.N., Fields S.D., Martin W., Qadota H., Benian G.M., Lu H., Barstead R.J. (2010). Alpha-actinin is required for the proper assembly of Z-disk/focal-adhesion-like structures and for efficient locomotion in Caenorhabditis elegans. J. Mol. Biol..

[55] Fyrberg C., Ketchum A., Ball E., Fyrberg E. (1998). Characterization of lethal Drosophila melanogaster alpha-actinin mutants. Biochem. Genet..

[56] Yang J., Xu X. (2012). Alpha-Actinin2 is required for the lateral alignment of Z discs and ventricular chamber enlargement during zebrafish cardiogenesis. FASEB J..

[57] Tanner B.C., Farman G.P., Irving T.C., Maughan D.W., Palmer B.M., Miller M.S. (2012). Thick-to-thin filament surface distance modulates cross-bridge kinetics in Drosophila flight muscle. Biophys. J..

[58] Martyn D., Chase P., Regnier M., Gordon A. (2002). A simple model with myofilament compliance predicts activation-dependent crossbridge kinetics in skinned skeletal fibers. Biophys. J..

[59] Williams C.D., Salcedo M.K., Irving T.C., Regnier M., Daniel T.L. (2013). The length-tension curve in muscle depends on lattice spacing. Proc. Biol. Sci..

[60] Foley K.S., Young P.W. (2014). The non-muscle functions of actinins: An update. Biochem. J..

[61] Sun B., Kekenes-Huskey P.M. (2023). Myofilament-associated proteins with intrinsic disorder (MAPIDs) and their resolution by computational modeling. Q. Rev. Biophys..

[62] Gregorio C.C., Trombitás K., Centner T., Kolmerer B., Stier G., Kunke K., Suzuki K., Obermayr F., Herrmann B., Granzier H. (1998). The NH2 terminus of titin spans the Z-disc: Its interaction with a novel 19-kD ligand (T-cap) is required for sarcomeric integrity. J. Cell Biol..

[63] Swist S., Unger A., Li Y., Vöge A., von Frieling-Salewsky M., Skärlén Å., Cacciani N., Braun T., Larsson L., Linke W.A. (2020). Maintenance of sarcomeric integrity in adult muscle cells crucially depends on Z-disc anchored titin. Nat. Commun..

[64] Hessel A.L., Ma W., Mazara N., Rice P.E., Nissen D., Gong H., Kuehn M., Irving T., Linke W.A. (2022). Titin force in muscle cells alters lattice order, thick and thin filament protein formation. Proc. Natl. Acad. Sci. USA.

[65] Risi C.M., Pepper I., Belknap B., Landim-Vieira M., White H.D., Dryden K., Pinto J.R., Chase P.B., Galkin V.E. (2021). The structure of the native cardiac thin filament at systolic Ca(2+) levels. Proc. Natl. Acad. Sci. USA.

[66] Risi C.M., Belknap B., White H.D., Dryden K., Pinto J.R., Chase P.B., Galkin V.E. (2023). High-resolution cryo-EM structure of the junction region of the native cardiac thin filament in relaxed state. PNAS Nexus.

[67] Veltri T., de Oliveira G.A.P., Bienkiewicz E.A., Palhano F.L., Marques M.A., Moraes A.H., Silva J.L., Sorenson M.M., Pinto J.R. (2017). Amide hydrogens reveal a temperature-dependent structural transition that enhances site-II Ca(2+)-binding affinity in a C-domain mutant of cardiac troponin C. Sci. Rep..

[68] Hancock W.O., Huntsman L.L., Gordon A.M. (1997). Models of calcium activation account for differences between skeletal and cardiac force redevelopment kinetics. J. Muscle Res. Cell Motil..

[69] Pinto J.R., Yang S.W., Hitz M.P., Parvatiyar M.S., Jones M.A., Liang J., Kokta V., Talajic M., Tremblay N., Jaeggi M. (2011). Fetal cardiac troponin isoforms rescue the increased Ca^2+^ sensitivity produced by a novel double deletion in cardiac troponin T linked to restrictive cardiomyopathy: A clinical, genetic, and functional approach. J. Biol. Chem..

[70] Johnston J.R., Landim-Vieira M., Marques M.A., de Oliveira G.A.P., Gonzalez-Martinez D., Moraes A.H., He H., Iqbal A., Wilnai Y., Birk E. (2019). The intrinsically disordered C terminus of troponin T binds to troponin C to modulate myocardial force generation. J. Biol. Chem..

[71] Fischetti R., Stepanov S., Rosenbaum G., Barrea R., Black E., Gore D., Heurich R., Kondrashkina E., Kropf A.J., Wang S. (2004). The BioCAT undulator beamline 18ID: A facility for biological non-crystalline diffraction and X-ray absorption spectroscopy at the Advanced Photon Source. J. Synchrotron. Radiat..

[72] Jiratrakanvong J., Shao J., Menendez M., Li X., Li J., Ma W., Agam G., Irving T. (2018). MuscleX: Software Suite for Diffraction X-ray Imaging, V1.13.1.

[73] Atkinson R.A., Joseph C., Kelly G., Muskett F.W., Frenkiel T.A., Nietlispach D., Pastore A. (2001). Ca^2+^-independent binding of an EF-hand domain to a novel motif in the alpha-actinin-titin complex. Nat. Struct. Biol..

[74] Pettersen E.F., Goddard T.D., Huang C.C., Couch G.S., Greenblatt D.M., Meng E.C., Ferrin T.E. (2004). UCSF Chimera--a visualization system for exploratory research and analysis. J. Comput. Chem..

[75] Sun B., Fang X., Johnson C.N., Hauck G., Kou Y., Davis J.P., Kekenes-Huskey P.M. (2021). Non-Canonical Interaction between Calmodulin and Calcineurin Contributes to the Differential Regulation of Plant-Derived Calmodulins on Calcineurin. J. Chem. Inf. Model.

[76] Maier J.A., Martinez C., Kasavajhala K., Wickstrom L., Hauser K.E., Simmerling C. (2015). ff14SB: Improving the Accuracy of Protein Side Chain and Backbone Parameters from ff99SB. J. Chem. Theory Comput..

[77] Jorgensen J.C.W.L., Madura J.D. (1983). Comparison of simple potential functions for simulating liquid water. J. Chem. Phys..

[78] Jean-Paul Ryckaert G.C., Berendsen H.J. (1977). C Numerical integration of the cartesian equations of motion of a system with constraints: Molecular dynamics of n-alkanes. J. Comput. Phys..

[79] Roe D.R., Cheatham T.E. (2013). PTRAJ and CPPTRAJ: Software for Processing and Analysis of Molecular Dynamics Trajectory Data. J. Chem. Theory Comput..

[80] Miller B.R., McGee T.D., Swails J.M., Homeyer N., Gohlke H., Roitberg A.E. (2012). MMPBSA.py: An Efficient Program for End-State Free Energy Calculations. J. Chem. Theory Comput..

