# Supplementary material for: Disruption of Z-Disc Function Promotes Mechanical Dysfunction in Human Myocardium: Evidence for a Dual Myofilament Modulatory Role by Alpha-Actinin 2
Source: Int J Mol Sci. 2023 Sep 26;24(19):14572. doi: 10.3390/ijms241914572 (PMC10572656; doi:10.3390/ijms241914572)
Supplement: Supplementary file 1 [file ijms-24-14572-s001.zip › ijms-2571469-supplementary.pdf]

SUPPLEMENTARY INFORMATION

Disruption of Z-disc function promotes mechanical dysfunction in human myocardium: evidence for a dual myofilament modulatory role by alpha-actinin 2.

Michelle Rodriguez Garcia, M.D.[1], Jeffrey Schmeckpeper, M.D., Ph.D.[2], Maicon Landim-Vieira, Ph.D.[1], Isabella Leite Coscarella, B.S [1]., Xuan Fang, Ph.D.[3], Weikang Ma, Ph.D.[4], Payton A. Spran, B.S.[5], Shengyao Yuan[4], Lin Qi[4], Aida Rahimi Kahmini, B.S.[6], M. Benjamin Shoemaker M.D., M.S.C.I.[2], James B. Atkinson, M.D., Ph.D.[7], Peter Kekenes-Huskey, Ph.D. [3], Thomas Irving, Ph.D.[4], Prescott Bryant Chase, Ph.D.[5], Bjorn Knollmann, M.D., Ph.D.[2] and Jose Renato Pinto, Ph.D.[1*]

Corresponding author: Jose R. Pinto

Email: jose.pinto@med.fsu.edu

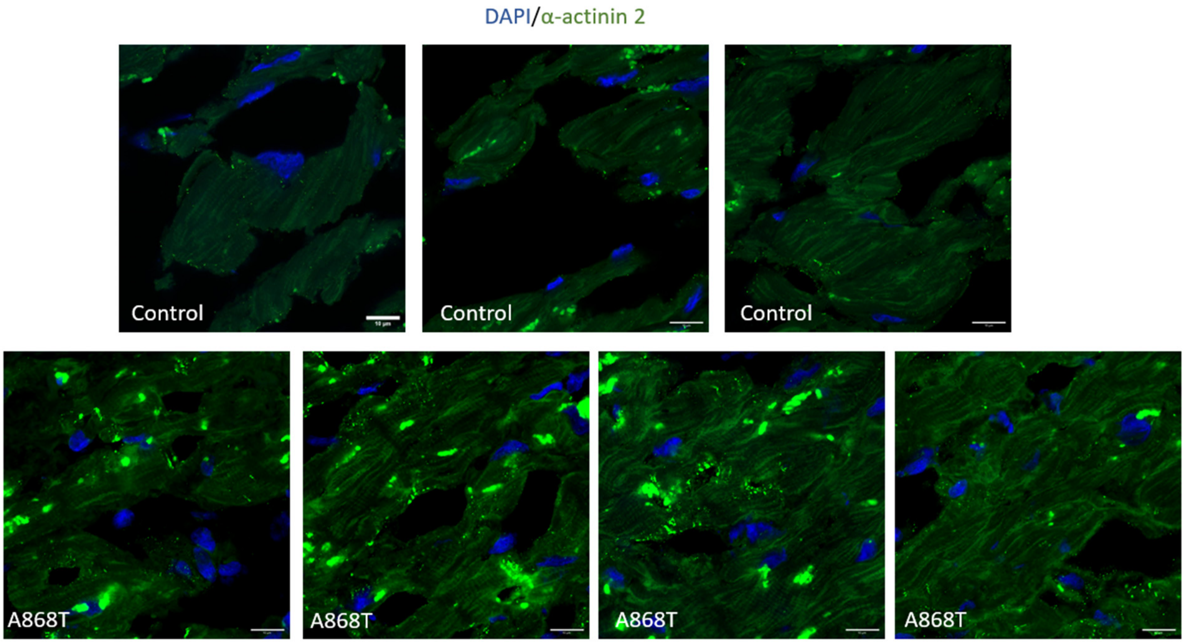

Supplemental Figure S1. Immunofluorescence assay. All the cryosectioned samples that were incubated with α-actinin 2 (GeneTex #GTX103219) antibody. Scale bars are 10 μm.

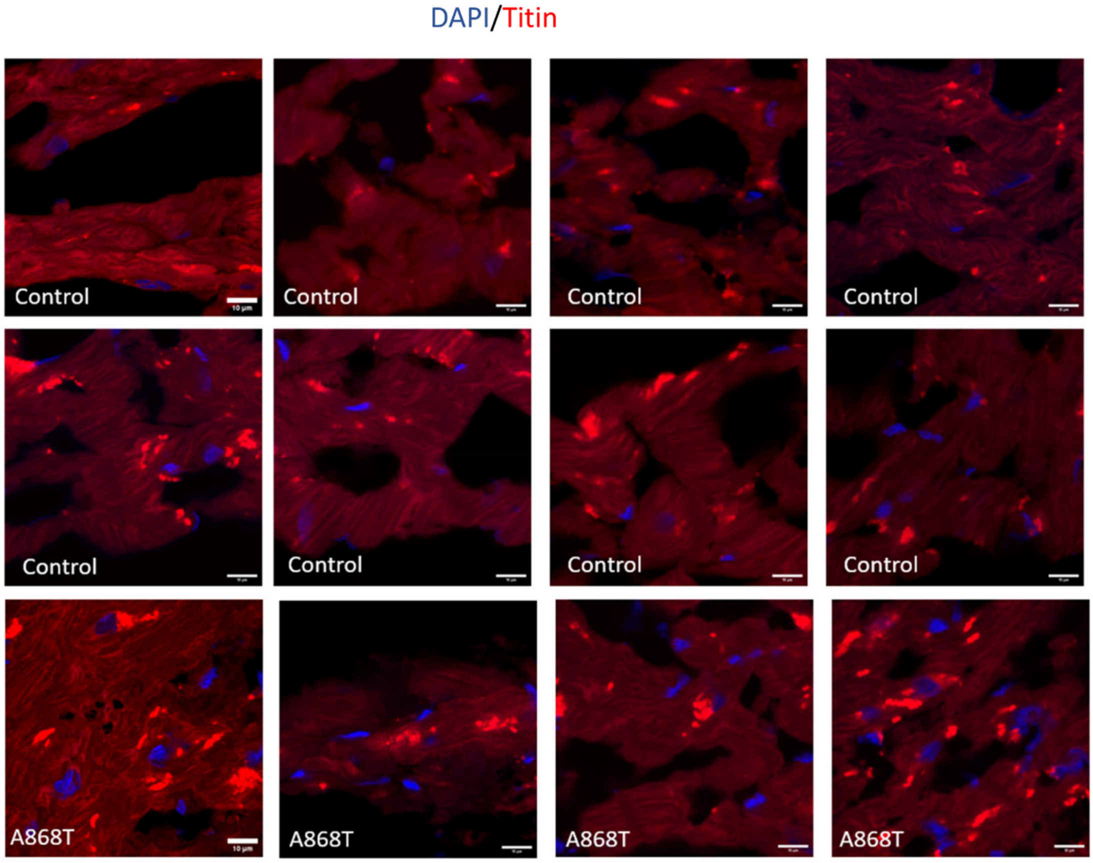

Supplemental Figure S2. Immunofluorescence assay. All the Cryosectioned samples that were incubated with Titin (Novus #NBP1-88071) antibody. Scale bars are 10 µm.

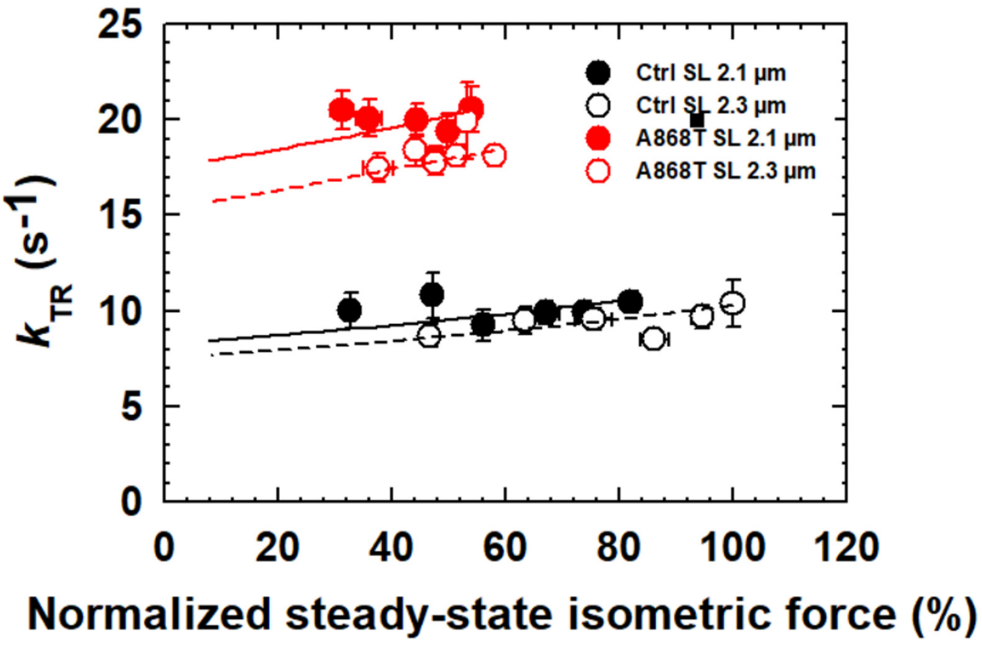

Supplemental Figure S3. Mathematical Modeling: Predictions of Kinetics of Tension Redevelopment as described in Gonzalez-Martinez et al.[1] The model proposes that *A868T* variant has faster rate of cross-bridges detachment *(g)* and slightly faster attachment *(f)* of cross bridges interaction in the sarcomere.

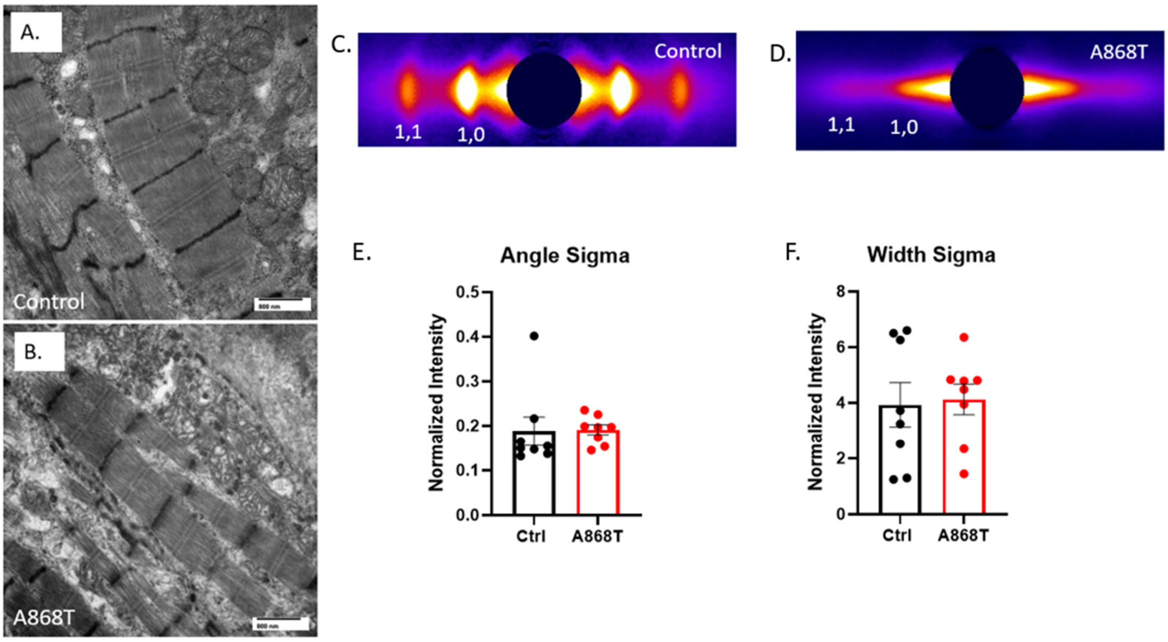

Supplemental Figure S4. Small-angle X-ray fiber diffraction: Angle and Width Sigma measurements. A-B) Electron Microscopy images where myofibril width was decreased. C-D) Small-angle X-ray fiber diffraction patterns showing a significantly larger lattice spacing (~10%) in the variant as compared to wild type. E-F). Angle sigma and width sigma were not significantly different implying that the degree of myofibrillar angular disarray and lattice spacing heterogeneity between myofibrils did not differ between the *A868T* variant and the control muscle.

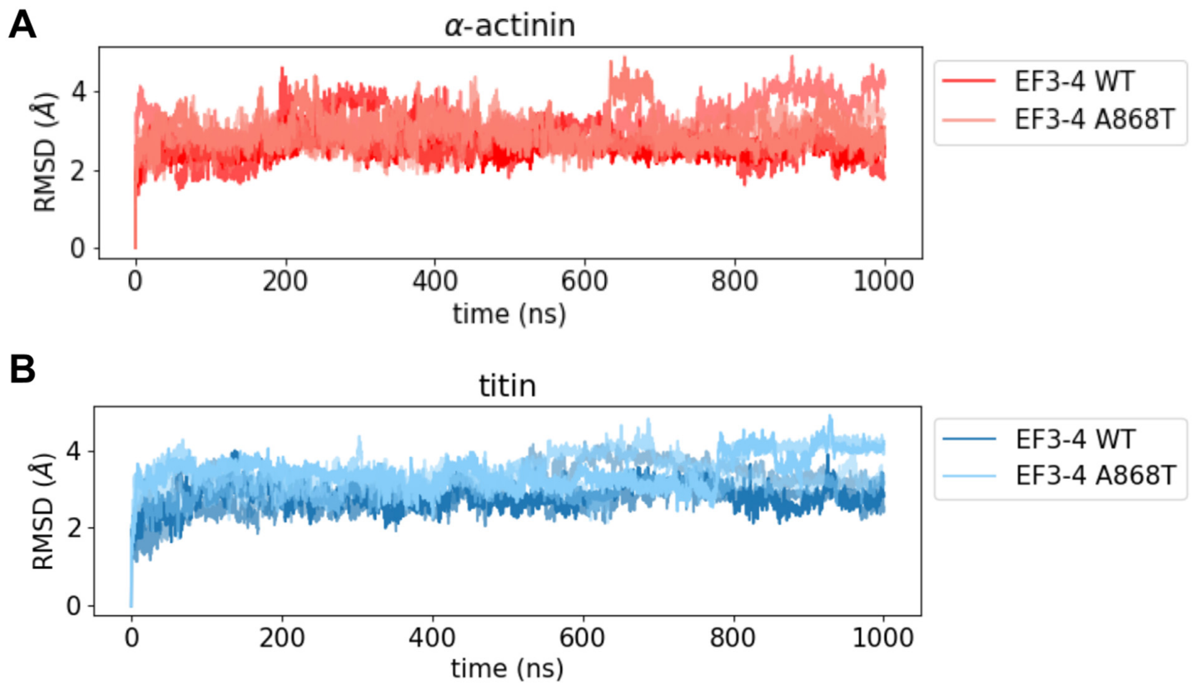

Supplemental Figure S5. RMSD of the EF3-4 - actinin (A) and - titin (B) systems. Both systems remained stable throughout the simulations. The first frame of each trajectory was used as the reference frame for calculation.

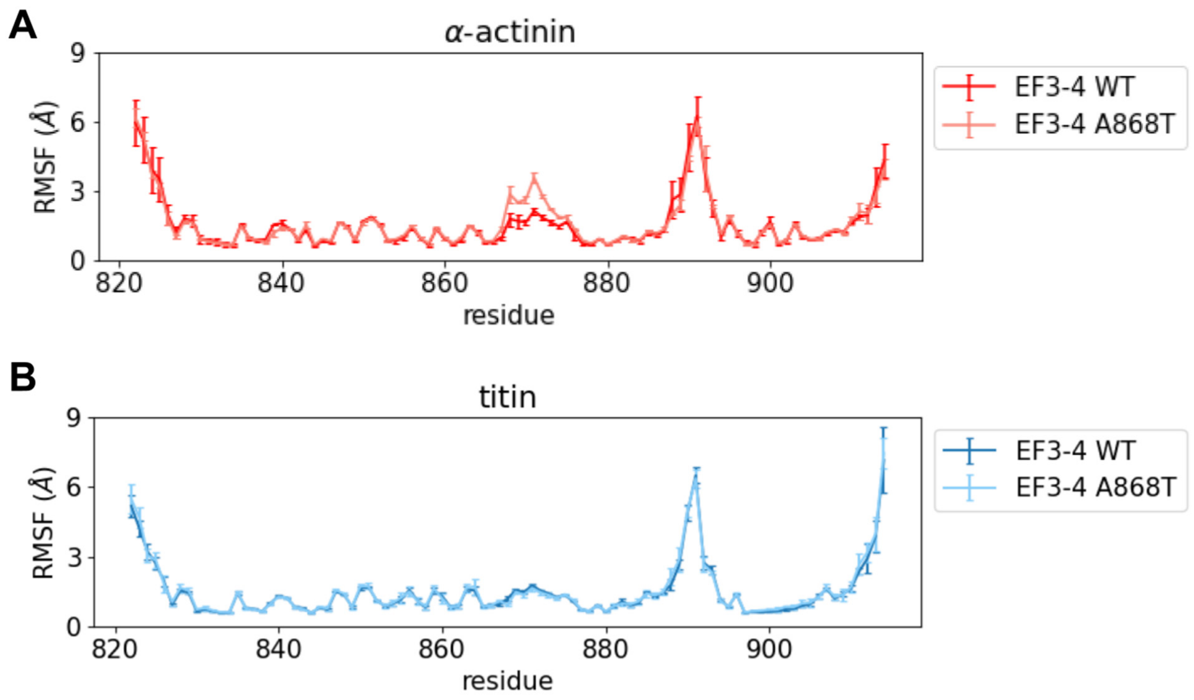

Supplemental Figure S6. RMSD of the EF3-4 - actinin (A) and - titin (B) systems. The actinin system showed differences near residue 50 between WT and mutant whereas WT and mutant of the titin system exhibited similar RMSF. The first frame of each trajectory was used as the reference frame for calculation.

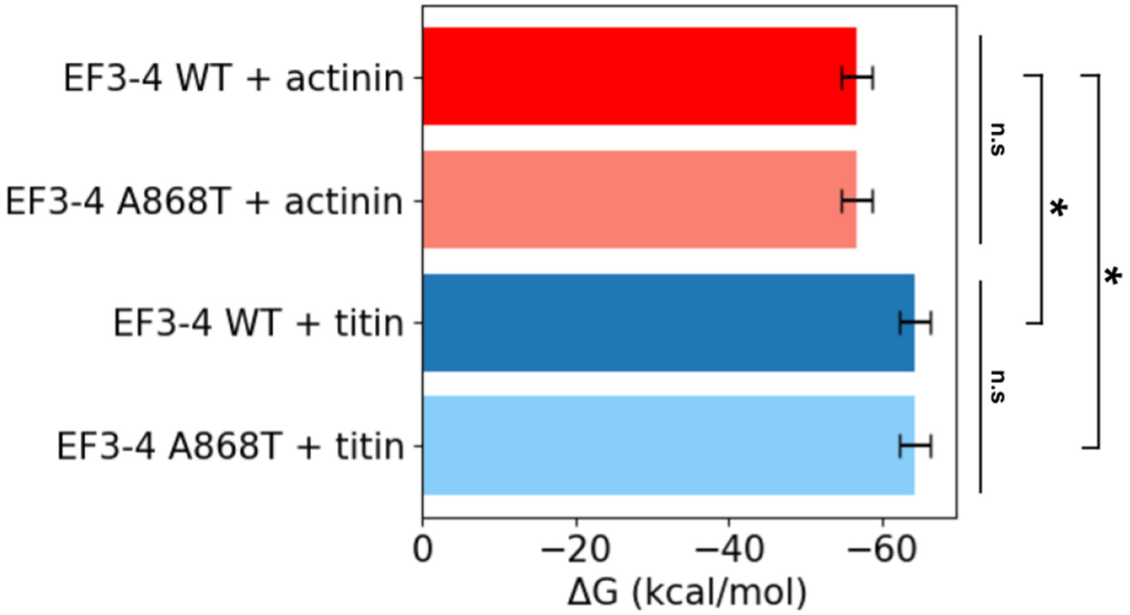

Supplemental Figure S7. Binding free energy computed by MM/GBSA. For each system, no significant difference was observed between WT and mutant. Across the two systems, however, EF3-4 showed more favorable binding to the titin peptide relative to the actinin neck region. Error bars represent the standard error of mean. * $p < 0.5$.

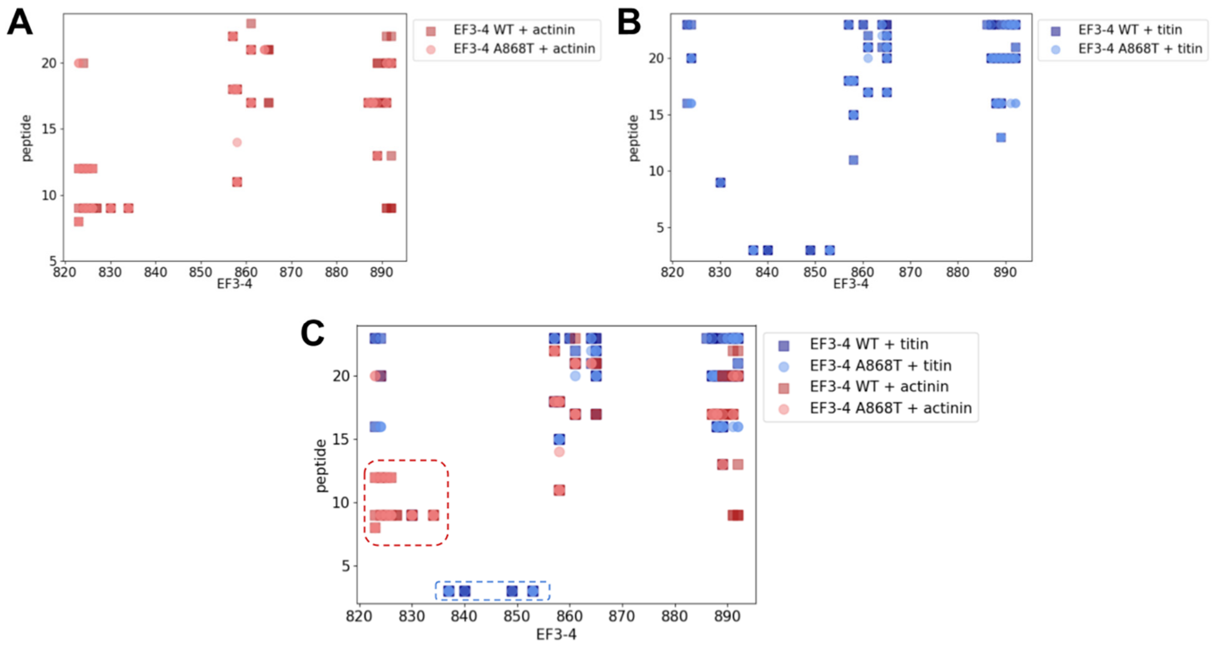

Supplemental Figure S8. Hydrogen bonding patterns in the EF3-4 - actinin (A), and - titin (B) system. C) Overlay of the hydrogen bonding network of the two systems showed unique patterns for each system.

	SL (µm)	Dextran T-500 (% wt:vol)	3-state model fitted parameters				3-state model predictions		
			Cross-bridge		Regulatory unit				
			f (s^{-1})	g (s^{-1})	k_{ON} (M^{-1} s^{-1}) (Pinto et al. 2011a)	k_{OFF} (s^{-1})	pCa_{50}	max force (norm)	max k_{TR} (s^{-1})
**WT**	2.3	3	2.8	7.6	1.84 x 10^8	402.0	5.81	1.0	10.3
**WT**	2.1	3	2.4	8.3	1.84 x 10^8	467.9	5.72	0.820	10.6
**ACTN2 A868T**	2.3	3	2.9	15.6	1.84 x 10^8	192.6	6.06	0.580	18.4
**ACTN2 A868T**	2.1	3	3.0	17.7	1.84 x 10^8	291.0	5.88	0.540	20.5

Isometric force normalized to control pCa 3.964, SL=2.3 mm

Supplemental Table S1. Mathematical Modeling: Kinetics of Tension Redevelopment. *A868T* CMPs displayed faster *g* (s^{-1}) in a 3-state modeling. All data was fitted to the isometric force of the Control (WT) at a SL of 2.3µm and pCa 3.964. CMPs (n) = 4-5 per group. Pinto et al. 2011a = PMID: 21056975

SI References

1. Gonzalez-Martinez, D., Johnston, J. R., Landim-Vieira, M., Ma, W., Antipova, O., Awan, O., Irving, T. C., Bryant Chase, P., and Pinto, J. R. (2018) Structural and functional impact of troponin C-mediated Ca(2+) sensitization on myofilament lattice spacing and cross-bridge mechanics in mouse cardiac muscle. *J Mol Cell Cardiol* **123**, 26-37
